# Structural diversity in binary superlattices self-assembled from polymer-grafted nanocrystals

**DOI:** 10.1038/ncomms10052

**Published:** 2015-12-02

**Authors:** Xingchen Ye, Chenhui Zhu, Peter Ercius, Shilpa N. Raja, Bo He, Matthew R. Jones, Matthew R. Hauwiller, Yi Liu, Ting Xu, A. Paul Alivisatos

**Affiliations:** 1 Department of Chemistry, University of California, Berkeley, California 94720, USA; 2 Advanced Light Source, Lawrence Berkeley National Laboratory, Berkeley, California 94720, USA; 3 National Center for Electron Microscopy, The Molecular Foundry, Lawrence Berkeley National Laboratory, Berkeley, California 94720, USA; 4 Department of Materials Science and Engineering, University of California, Berkeley, California 94720, USA; 5 Materials Sciences Division, Lawrence Berkeley National Laboratory, Berkeley, California 94720, USA; 6 The Molecular Foundry, Lawrence Berkeley National Laboratory, Berkeley, California 94720, USA; 7 Kavli Energy NanoScience Institute, Berkeley, California 94720, USA

## Abstract

Multicomponent nanocrystal superlattices represent an interesting class of material that derives emergent properties from mesoscale structure, yet their programmability can be limited by the alkyl-chain-based ligands decorating the surfaces of the constituent nanocrystals. Polymeric ligands offer distinct advantages, as they allow for more precise tuning of the effective size and ‘interaction softness' through changes to the polymer's molecular weight, chemical nature, architecture, persistence length and surrounding solvent. Here we show the formation of 10 different binary nanocrystal superlattices (BNSLs) with both two- and three-dimensional order through independent adjustment of the core size of spherical nanocrystals and the molecular weight of densely grafted polystyrene ligands. These polymer-brush-based ligands introduce new energetic contributions to the interparticle potential that stabilizes various BNSL phases across a range of length scales and interparticle spacings. Our study opens the door for nanocrystals to become modular elements in the design of functional particle brush solids with controlled nanoscale interfaces and mesostructures.

## Abstract (web summary)

Binary nanocrystal superlattice metamaterials are arousing significant interest due to their potential for use in functional devices. Here, the authors endow the nanoparticles with polymer brushes which enable control over their spacings and thus mesoscale structure and properties.

## Graphical abstract

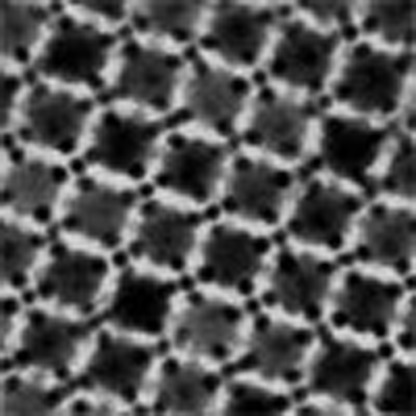

Ordered arrays of colloidal nanocrystals (NCs) represent an important class of metamaterials with tunable structures and properties^1^,^2^,^3^,^4^,^5^,^6^,^7^,^8^,^9^. Progress in NC synthesis and self-assembly has enabled co-crystallization of two types of NCs into binary nanocrystal superlattices (BNSLs) exhibiting a wide range of stoichiometries and lattice symmetries^4^,^8^,^10^,^11^,^12^,^13^,^14^. The vast majority of studies on BNSLs thus far involve the self-assembly of colloidal NCs that are capped with pristine alkyl ligands^4^,^10^,^11^,^12^,^14^. As a consequence, the structural tunability in BNSLs is often intimately connected to one's ability to synthetically adjust the inorganic core size of the NCs^4^. However, it is not always feasible to tune the NC size across a broad range for a given material. More importantly, the physical properties of NCs exhibit a strong dependence on their dimensionality^1^. To harness the full potential of self-assembly as a route to rationally designed metamaterials and functional devices, it is necessary to develop a modular building block in which the inorganic core and the organic ligand shell play complementary and equally important roles in dictating the structure and function of the final assembled phase.

Polymer brushes, or polymer chains end-tethered to a surface through covalent or coordinative interactions, represent an interesting class of soft materials^15^,^16^,^17^,^18^,^19^,^20^,^21^,^22^,^23^,^24^. The conformational flexibility and functional versatility inherent to these systems make polymer brushes an attractive platform for many applications in nanotechnology and biotechnology^20^,^24^,^25^,^26^,^27^. More importantly, grafting polymers onto NCs introduces new energetic contributions arising from polymer chain conformational entropy and interaction enthalpy. These energetic contributions can be readily tailored by the control of the polymer's molecular weight, chemical nature, architecture, persistence length and surrounding solvent. Therefore, the incorporation of polymeric ligands would greatly expand the parameter space for the design of ordered particle brush solids. Over the past several decades, extensive experimental and theoretical efforts have been devoted to studies of various particle-polymer brush hybrid materials. Several recent examples of self-organization of polymer-grafted NCs into ordered assemblies have demonstrated the formation of single-component two-dimensional (2D) hexagonal lattices^18^,^19^,^25^,^28^,^29^,^30^,^31^,^32^,^33^,^34^. In contrast, limited success has been achieved in creating more complex, binary arrays of polymer-grafted NCs with both two- and three-dimensional (3D) order. In one notable example, the Bockstaller group has studied binary mixtures of polystyrene-coated Au NCs and silica nanoparticles, and observed that the small Au NCs segregate preferentially to interstitial sites within a regular array formed by the larger silica nanoparticles^35^.

In this work, we explore the self-assembly of binary mixtures of polymer-grafted NCs into long-range-ordered particle brush solids. We carry out detailed studies of the polymer brush characteristics on a wide variety of NCs, in both solvated and dried states. These results allow us to access a set of modular building blocks from which a desired BNSL phase can be created via rational choice of NC component. Distinct from alkyl-chain-based ligands, one unique advantage of using polymeric ligands is that the interparticle spacing can be predicted and experimentally accessed with high precision ranging from one to several tens of nanometres, which can be important for tailoring magnetic dipolar coupling^36^, plasmonic coupling^37^,^38^ and energy transfer mediated by dipole–dipole interactions within NC assemblies^39^.

## Results

### Synthesis and characterization of polymer-grafted NCs

To introduce polymeric ligands onto the surfaces of NCs, we used the ‘grafting-to' method, as it allows for independent optimization of the synthesis of inorganic NCs and polymers. Monodisperse Au and Fe_3_O_4_ NCs were first synthesized according to established protocols (Supplementary Fig. 1)^40^,^41^,^42^. We then carried out a simple ligand-exchange process to graft a dense layer of low-polydispersity polymers to the NCs (Supplementary Table 1). We chose atactic polystyrene, an amorphous polymer that is often regarded as a model polymer system^21^,^27^,^30^,^34^,^35^,^43^,^44^,^45^,^46^. For Au NCs, thiol-terminated polystyrene was used to replace the pristine oleylamine ligand, given the strong affinity between thiol groups and gold surfaces. On the other hand, carboxyl-terminated polystyrene was employed to displace the native oleic acid ligands of Fe_3_O_4_ NCs, driven largely by the law of mass action. Following ligand exchange, it was found that both Au and Fe_3_O_4_ NCs became insoluble in hexane but were readily soluble in toluene, chloroform and tetrahydrofuran, all of which are known to be good solvents for polystyrene. The ligand exchange was also monitored using Fourier transform infrared (FTIR) spectroscopy. Distinct vibrational fingerprints of the aromatic moiety were evident for all polystyrene-exchanged NCs, but were noticeably absent for the as-synthesized NCs (Supplementary Fig. 2)^47^. Altogether, these results indicate the presence of an appreciable amount of polystyrene on the surfaces of the NCs.

To probe more quantitatively the ligand-exchange reaction, thermal gravimetric analysis (TGA) was used to evaluate the ligand grafting density. For polymer-grafted NCs, weight loss events were observed at elevated temperatures (above 250 °C), originating predominantly from thermal degradation of polystyrene chains into volatile products composed mostly of styrene monomer and oligomers (Supplementary Fig. 3)^48^. As summarized in Table 1, there appears to be a clear trend of decreasing grafting densities with increasing polystyrene chain lengths for Au NCs of various core sizes. This may be qualitatively understood using de Gennes's scaling model for a polymer chain confined inside a small cylindrical tube: the loss in conformational entropy for constrained polystyrene chains can be more than compensated for by the enthalpy gain associated with bond formation between the polymer anchoring groups (thiols or carboxylic acids) and the NC surfaces^49^. As the enthalpic contribution remains unchanged regardless of the polystyrene chain length, grafting a longer chain will become energetically more costly unless the chain can be confined within a tube of greater diameter. In addition, the dynamic, self-limiting nature of the ligand-exchange process may also play a role. The already-grafted polymer chains can introduce a steric barrier for subsequent chains to reach the binding sites on the NC surface. This effect can be more pronounced for longer chains. From the TGA-derived grafting densities, we estimated the average distance between adjacent ligand anchoring sites by assuming that the effective footprint of individual ligand molecules is a circular area with a diameter equal to the distance between adjacent chains. As shown in Supplementary Table 2, the average spacings between polystyrene ligands on Au NCs are considerably smaller than the Flory radius *R*_F_, defined as the root-mean-squared end-to-end distance for a free polymer chain^50^. Therefore, the polystyrene chains are expected to extend from the NC surface much farther than their natural dimensions to avoid steric overcrowding, thus forming a polymer ‘brush'. Moreover, the grafting densities on 3.8 nm (core diameter) Au NCs are consistently larger than those on 7.6 nm Au NCs for the same polystyrene chain length, which may be attributed to the fact that the smaller radius of curvature of larger NCs can create a more crowded local environment for the polymer chains.

### Scaling behaviour of polymer brushes on spherical NCs

The development of polymer brush theories dates back to the 1970s. In their pioneering work, Alexander^51^ and de Gennes^52^ theoretically predicted that the thickness of a planar polymer brush in a good solvent scales as *h*∼*Nσ*^1/3^, where *N* is the degree of polymerization for the grafted polymer and *σ* is the grafting density. Distinct from planar brushes, a spherical interface implies that the effective ligand areal density is not invariant but decreases with increasing radial distance from the surface. Therefore, one would expect drastically different scaling behaviour between spherical and planar polymer brushes.

Daoud and Cotton adopted the Alexander and de Gennes's free energy scaling approach and developed scaling models for star-shaped polymers^53^. The widely used Daoud–Cotton model set up a reasonable theoretical framework for the understanding of spherical polymer brushes and polymers tethered to curved surfaces. Later, Birshtein and Zhulina determined that the thickness of a polymer brush layer grafted to a spherical surface in the melt state (that is, in the absence of solvent) scales as *h*_*θ*_∼*N*^1/2^*σ*^1/4^, and that in a good solvent scales as *h*∼*N*^3/5^*σ*^1/5^ (refs 54, 55, 56). These different scaling laws highlight the dissimilarity of the mechanisms underlying polymer chain conformations between the solvated and the dried state. In the presence of solvent, polymer chains stretch away from the grafting surface by virtue of the effective repulsions between chain segments. In the dried state, however, the chains stretch away from the interface to alleviate steric constraints or overfilling of space.

**Figure 1:**
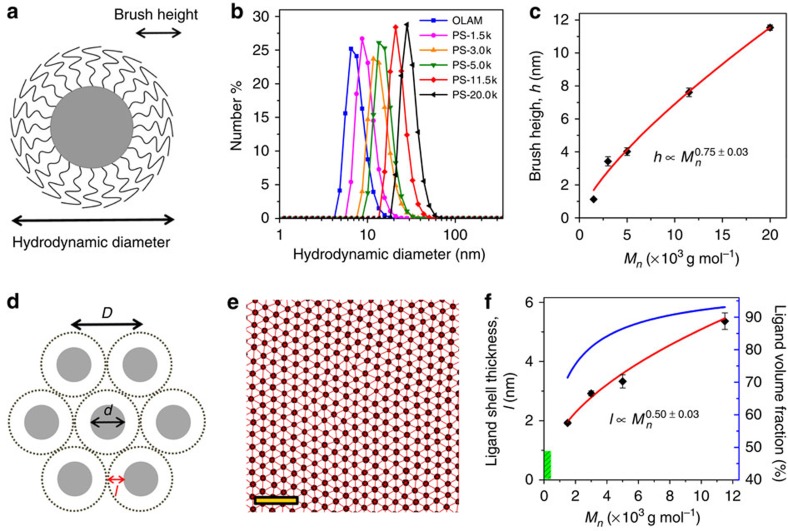
Scaling behaviour of polymer brushes on spherical NCs. (**a**) Schematic illustration of NCs end-grafted with polymer brushes in good solvents. (**b**) Plots of number-averaged hydrodynamic diameter and (**c**) scaling relation of DLS-derived brush height, *h*, as a function of the molecular weight of thiol-terminated polystyrene ligands for 7.6 nm Au NCs dispersed in toluene. (**d**) Schematic showing the relationship between *D*, the centre-to-centre distance between nearest-neighbour NCs, *d*, the diameter of the inorganic core of the NCs, and the ligand shell thickness, *l=*(*D−d*)/2, for two-dimensional hexagonal NC superlattices. (**e**) Particle tracking analysis of TEM images for statistical determination of average nearest-neighbour interparticle distance *D*. A sample area of two-dimensional assemblies of 7.6 nm Au NCs is shown. Scale bar, 50 nm. (**f**) Scaling relations of ligand shell thickness *l*, and calculated ligand volume fraction (blue curve) as a function of molecular weight for 7.6 nm Au NCs in the dried state. The green area highlights the range of *M*_*n*_ and *l* accessible by using commonly employed alkyl ligands. The ligand volume fraction is computed as 
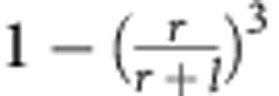
, where *r=d*/2. The red curves in **c** and **f** represent the best fit to the data using a power-law function.

Spherical polymer brushes represent a model system that is intermediate between flat brushes and star-shaped polymers. A linear polymer chain attached by one end to a spherical NC at relatively high coverage is expected to take on more stretched conformations in the vicinity of NC surface and assume progressively more relaxed configurations with increasing radial distance from the NC surface, as pictured schematically in Fig. 1a. Polymer brushes grafted onto spherical NCs dispersed in a good solvent can be categorized into three different regimes depending on the grafting density and the polymer chain length^26^,^31^,^32^,^33^,^57^. At low grafting densities, polymer chains adopt an approximately random coil conformation, the so-called ‘mushroom' regime. As the grafting density exceeds the chain overlap threshold, a transition to the semidilute polymer brush regime takes place, resulting in the stretching of polymer chains normal to the grafting surface. In the limit of high grafting densities, the concentrated polymer brush (CPB) regime is accessed, which is characterized by non-Gaussian chain characteristics and more extended chain conformations due to excluded volume interactions. Dukes *et al.*^57^ concluded that for a spherically symmetric grafting surface the hydrodynamic thickness of polymer brushes scale as *h*∼*N*^4/5^ for the CPB regime but *h*∼*N*^3/5^ for the semidilute polymer brush regime. These predictions have been shown to be broadly applicable to many spherical NC-polymer brush systems.

Here we used dynamic light scattering (DLS) to probe the solvent-swollen polymer brush thickness of functionalized spherical Au and Fe_3_O_4_ NCs. As shown in Fig. 1b and Supplementary Fig. 4, DLS measurements revealed a relatively narrow size distribution (coefficient of variation <7%) for NCs before and after ligand exchange. The average hydrodynamic diameter of Au NCs was found to increase monotonically with increasing molecular weight of polystyrene for all three NC core sizes studied, providing direct evidence for the successful and homogeneous attachment of polystyrene chains at the ensemble level. The height of the solvated polymer brushes *h* can be calculated by subtracting the NC core radius determined by transmission electron microscopy (TEM) image analysis from the hydrodynamic radius retrieved from DLS measurements (Supplementary Tables 3 and 4). As shown in Fig. 1c, the scaling relation between *h* and the molecular weight *M*_*n*_ of polystyrene ligands grafted onto 7.6 nm Au NCs can be well described by the power law 
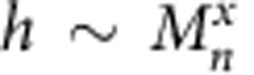
, with the scaling exponent *x* to be about 0.75. Similar scaling relations with exponents between 0.7 and 0.8 were obtained for 3.8 and 4.5 nm Au NCs (Supplementary Fig. 4). These scaling results indicate that it is appropriate to consider the polystyrene chains on Au NCs to be in the CPB regime^26^,^31^,^32^,^33^,^57^.

It is also instructive to assess the scaling relation between the polymer-brush thickness and the molecular weight of polystyrene *M*_*n*_ in the melt. The interparticle spacing in a ‘dried' NC array is largely determined by the ligand chain configuration as well as degree of chain interdigitation between adjacent NCs. The ligand layer thickness *l*, estimated as half of the nearest-neighbour surface-to-surface interparticle separation in 2D NC superlattices, serves as a good measure of the size of the tethered polymer chains (Fig. 1d). The average thickness of the ligand layer, determined from statistical analysis of a large set of nearest-neighbour NC pairs from TEM images (Fig. 1e and Supplementary Figs 5 and 6), was found to increase systematically with increasing molecular weight of the polystyrene ligand (Supplementary Tables 5 and 6). Well-defined interparticle spacings with a narrow distribution (coefficient of variation <8%) are observed for all 2D NC superlattices (Supplementary Fig. 6), providing compelling evidence for the presence of homogeneously dense polymer brushes on the NC surfaces. It is found that the brush thickness scales roughly as the square root of the molecular weight of the polymer chains, which is almost independent of the core size of Au NCs (Fig. 1f, red curve and Supplementary Fig. 7). These results are in line with the above-mentioned theoretical predictions as well as recent experimental findings on polymer brushes bound to spherical surfaces^25^,^54^,^55^,^56^,^57^. Remarkably, the polystyrene ligands take up at least 70% of the total volume within these hybrid building blocks (Fig. 1f, blue curve). Compared with commonly employed alkyl ligands, the range of accessible ligand shell thickness can be greatly expanded by using polymeric ligands. It is worth pointing out that even though the observed scaling behaviour is similar to unperturbed polymer chains in the melt, the polystyrene chains on NCs are indeed stretched. The average ligand layer thickness is found to be about twice the radius of gyration (*R*_*g*_) of free polystyrene chains (Supplementary Table 6), which seems somewhat contradictory with the conclusions drawn from TGA or DLS measurements that the polystyrene chains are stretched and can be treated as polymer brushes. These results can be reconciled by considering that the areal ligand density on the outer NC surface can be reduced appreciably by a factor of 
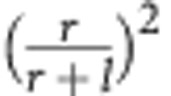
compared with the grafting density, providing room for polystyrene chains from approaching NCs to interpenetrate. We note that the polymer chains can also deform and fill the interstitial space in NC superlattices. Interpenetration between spherical polymer brushes and polymer chain deformation help reduce the energetically unfavourable polymer-air interfacial area and act to densify NC packing^14^,^35^.

### Three-dimensional binary superlattices

Using these monodisperse polystyrene-capped Au and Fe_3_O_4_ NCs as spherical building blocks, we explore the possibility of order formation in a binary mixture of NC polymer brushes. BNSLs were formed by slow drying of a toluene solution of mixed NCs on top of an immiscible liquid subphase, namely, diethylene glycol (DEG)^12^. Fe_3_O_4_ NCs (13.4 nm) functionalized with polystyrene having a number-average molecular weight *M*_*n*_=5.3 k, denoted as 13.4 nm (5.3 k) Fe_3_O_4_ NCs, were chosen throughout this work as the large NC building blocks for BNSLs. The experimentally derived polymer brush scaling relations or ‘master curves' for different-sized Au NCs allow for modular design of smaller NC building blocks targeting a specific NC size ratio (Fig. 1f, red curve and Supplementary Fig. 7).

**Figure 2:**
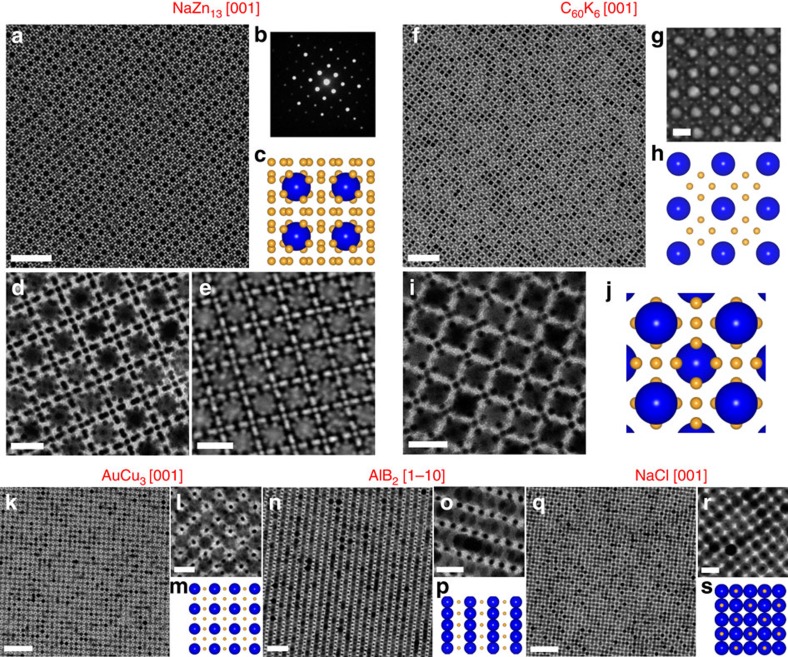
Structural diversity in 3D BNSLs self-assembled from 3.8 nm (3.0 k) Au and 13.4 nm (5.3 k) Fe_3_O_4_ NCs. (**a**) Low-magnification TEM image and (**b**) corresponding SAED pattern, (**d**) high-magnification TEM image and (**e**) HAADF-STEM image of NaZn_13_-type BNSLs. (**c**) Structural model of the [001] projection of NaZn_13_-type BNSLs. (**f**) Low-magnification TEM image, (**i**) high-magnification TEM image and (**g**) SEM image of bcc-AB_6_-type (isostructural with the C_60_K_6_ phase) BNSLs. (**h**,**j**) Structural models of the [001] projection (**j**) and the (001) surface (**h**) of bcc-AB_6_-type BNSLs. (**k**,**n**,**q**) Low-magnification TEM images, (**l**,**o**,**r**) high-magnification TEM images and (**m**,**p**,**s**) structural models of AuCu_3_-type (**k**–**m**), AlB_2_-type (**n**–**p**) and NaCl-type (**q**–**s**) BNSLs. Scale bars, (**a**) 100 nm; (**d**) 20 nm; (**e**) 20 nm; (**f**) 100 nm; (**g**) 20 nm; (**i**) 20 nm; (**k**) 100 nm; (**l**) 20 nm; (**n**) 50 nm; (**o**) 20 nm; (**q**) 100 nm; (**r**) 20 nm.

Figure 2a shows a representative TEM image of NaZn_13_-type BNSLs self-assembled from 3.8 nm (3.0 k) Au and 13.4 nm (5.3 k) Fe_3_O_4_ NCs. Both the cubic symmetry and the long-range positional ordering are clearly manifested by the sharp diffraction spots in the selected-area electron diffraction (SAED) pattern recorded from the same BNSL domain (Fig. 2b). High-magnification TEM imaging (Fig. 2d) and high-angle annular dark field scanning TEM (HAADF-STEM) imaging (Fig. 2e) match the [001] projection of the NaZn_13_ structure in which (Au)_13_ icosahedral clusters are separated by simple cubic arrangements of Fe_3_O_4_ NCs (Fig. 2c and Supplementary Figs 8 and 9)^58^. Importantly, a greater portion of electron-transparent regions within these assemblies are observed in comparison to those BNSLs formed by NCs capped with short alkyl chains^4^,^11^,^12^,^14^,^36^,^58^, highlighting the presence of dense polystyrene brushes on the NC surfaces. Low-magnification TEM image reveals a mosaic texture of predominantly [001]-oriented BNSL grains separated by narrow, glassy regions (Supplementary Fig. 10). The 3.8 nm (3.0 k) Au NC has an average ligand shell thickness *l*=3.0 nm (Supplementary Table 6), exceeding its core radius (1.9 nm), whereas *l*=3.3 nm for the 13.4 nm (5.3 k) Fe_3_O_4_ NC. The effective NC radius is computed as the sum of NC core radius and the ligand shell thickness, allowing the size ratio *γ* (ratio of effective radius between small and large NCs) to be readily calculated to be *γ*=0.49.

To study the formation of other BNSL structures using the same combination of 3.8 nm (3.0 k) Au and 13.4 nm (5.3 k) Fe_3_O_4_ NCs, we systematically varied the concentration ratio of the two NC building blocks. When the NC concentration ratio is reduced from ∼15:1 used for NaZn_13_ to ∼7:1, bcc-AB_6_-type BNLSs were obtained. The bcc-AB_6_ structure was initially identified in several alkali-metal intercalation compounds of fullerene C_60_ such as C_60_K_6_ and C_60_Cs_6_ (ref. 59). The unit cell is composed of a body-centred cubic (BCC) lattice formed by the large spheres, with four small spheres arranged into a square on each of the six facets of the cube (Supplementary Fig. 8). Recently, it has been observed in assemblies of oppositely charged micron-sized colloids^60^, BNSLs^14^,^41^ and binary lattices of DNA-coated particles^8^,^61^. Figure 2f,i shows representative TEM images of a [001]-oriented domain of the bcc-AB_6_-type BNSLs. Scanning electron microscopy (SEM) was exploited to probe the surface structure of the bcc-AB_6_-type BNSLs. As shown in Fig. 2g and Supplementary Fig. 11, the arrangements of NCs on the top layers match the structural model of the (001) plane, confirming the structural assignment as bcc-AB_6_. Further decreases in NC concentration ratio result in the formation of phase-pure BNSLs of lower stoichiometries, including AuCu_3_ (Fig. 2k–m and Supplementary Fig. 12a), AlB_2_ (Fig. 2n–p and Supplementary Fig. 12b) and NaCl (Fig. 2q–s). It is worth emphasizing that although these structures have been observed in earlier works, this is the first demonstration of the ability to access all five BNSL structures using NCs with a single size ratio (*γ*=0.49)^4^,^14^. In other words, these polystyrene-grafted NCs appear to be more ‘responsive' to changes in the NC mixing ratio during BNSL self-assembly.

**Figure 3:**
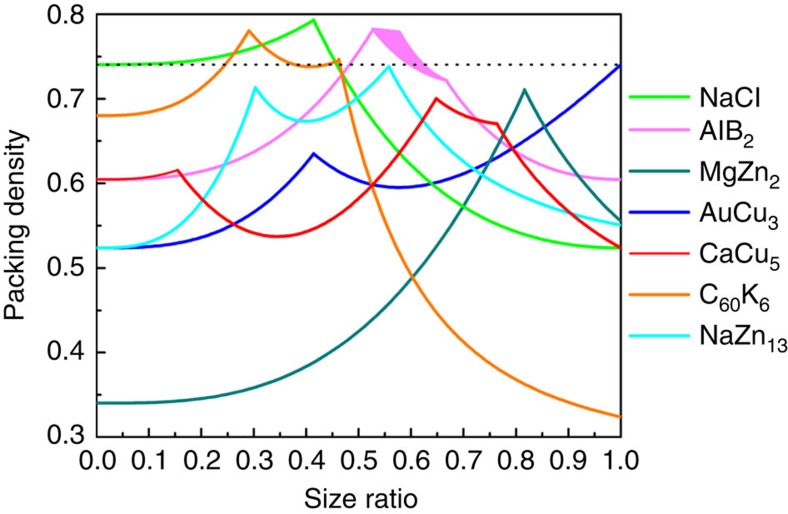
Theoretical packing densities for different BNSL phases. Plots of hard-sphere packing density versus size ratio for various BNSL phases reported in this work. The horizontal dashed line represents the packing density *ρ*=0.7405 for single-component close-packed structures.

The crystallization mechanism of BNSLs has been largely attributed to entropic driving forces^13^,^41^,^58^, although complex interparticle interactions such as van der Waals, dipolar, electrostatic, and so on, have also been implicated^4^,^11^. Recently, many-body effects and deformability of the ligand shell have been invoked to explain the entropy-driven crystallization of soft nanoscale objects^14^. Despite these conceptual advances aimed at improved understanding of BNSL formation, phase stability analysis based upon a hard sphere approximation (that is, the space-filling principle) still provides a reasonable theoretical framework and useful experimental guideline. This is due primarily to the fact that this model helps to rationalize the relative stability of plausible BNSL phases for a given effective size ratio or the range of size ratios needed to form a particular BNSL structure. Theoretical packing densities as a function of particle size ratio can be derived by modelling BNSL structures as sphere packings (Fig. 3)^11^,^14^,^41^. It is clear that the majority of BNSLs (except the AuCu_3_ structure) obtained with polystyrene-grafted NCs at the size ratio *γ*=0.49 feature a reasonably high packing density (≥0.67).

**Figure 4:**
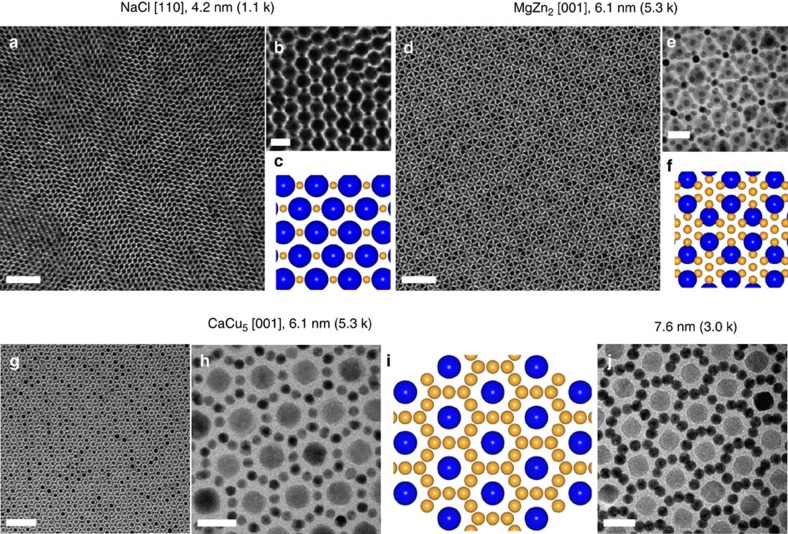
Tuning BNSL structures through variations of NC core size and the chain length of polymer brushes. (**a**) Low-magnification and (**b**) high-magnification TEM images of NaCl-type BNSLs self-assembled from 4.2 nm (1.1 k) Au and 13.4 nm (5.3 k) Fe_3_O_4_ NCs. (**c**) Structural model of the [110] projection of NaCl-type BNSLs. (**d**) Low-magnification and (**e**) high-magnification TEM images of MgZn_2_-type BNSLs self-assembled from 6.1 nm (5.3 k) Au and 13.4 nm (5.3 k) Fe_3_O_4_ NCs. (**f**) Structural model of the [001] projection of MgZn_2_-type BNSLs. (**g**) Low-magnification and (**h**) high-magnification TEM images of CaCu_5_-type BNSLs self-assembled from 6.1 nm (5.3 k) Au and 13.4 nm (5.3 k) Fe_3_O_4_ NCs. (**i**) Structural model of the [001] projection of CaCu_5_-type BNSLs. (**j**) TEM image of CaCu_5_-type BNSLs self-assembled from 7.6 nm (3.0k) Au and 13.4 nm (5.3 k) Fe_3_O_4_ NCs. Scale bars, (**a**) 100 nm; (**b**) 20 nm; (**d**) 100 nm; (**e**) 20 nm; (**g**) 100 nm; (**h**) 20 nm; (**j**) 20 nm.

The structure of BNSL assemblies can also be adjusted by tuning the size ratio *γ*. As shown in Fig. 4a–c, NaCl-type BNSLs were predominantly observed when using the combination of 4.2 nm (1.1 k) Au and 13.4 nm (5.3 k) Fe_3_O_4_ NCs, for which *γ*=0.40. This can be accounted for by the fact that the NaCl structure features a theoretical packing density *ρ*=0.793 that far exceeds other BNSL phases at this size ratio (Fig. 3). On the other hand, MgZn_2_-type (Fig. 4d–f) and CaCu_5_-type BNSLs (Fig. 4g–i) became the preferred structures at *γ*=0.69 when 6.1 nm (5.3 k) Au was employed as the smaller NC component. Both structures show significantly increased packing densities when *γ* increases from 0.49 to 0.69 (Fig. 3). Remarkably, the same CaCu_5_-type BNSLs was obtained by using Au NCs having a larger core size (7.6 nm) yet capped with shorter polystyrene chains (*γ*=0.68), highlighting the versatility of using spherical polymer-grafted NCs as modular building blocks for constructing multicomponent assemblies (Fig. 4j).

The presence of polymeric grafts in the CPB regime on NCs can have complex and intriguing implications on the interparticle interaction potential^62^,^63^. In good solvents, the highly extended polymer chains can effectively screen the van der Waals attractions between NC cores and exert steric interactions over a longer range than previously used short alkyl chain ligands. During the late stages of drying-mediated assembly, polymer brushes become compressed but can still penetrate into the outer layers of opposing brushes, allowing for a softening of the repulsive interactions between the NCs. The interplay between polymer chain stretching and chain interpenetration maximizes the overall entropy of the system during the formation of superlattices. Moreover, the softness of the interaction potential can be programmed by varying the thickness of the polymer brushes. For single-component NC assemblies, we indeed observed a structural transition from close-packed structures (fcc or hcp) to a BCC lattice as the size ratio between the polymer ligand and NC core increased (Supplementary Fig. 13). Similar trends have been observed in other soft colloidal systems such as block copolymer micelles^64^ and NCs^65^,^66^.

### Two-dimensional binary superlattices

**Figure 5:**
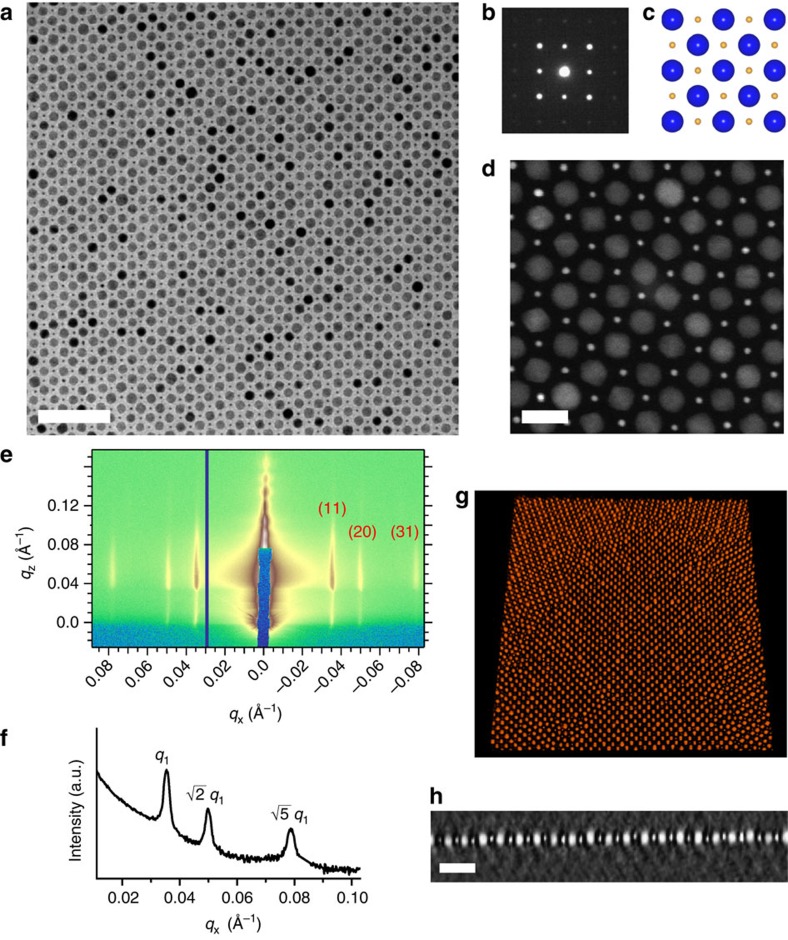
Two-dimensional BNSLs self-assembled from 3.8 nm (3.0 k) Au and 13.4 nm (5.3 k) Fe_3_O_4_ NCs. (**a**) Low-magnification TEM image and (**b**) corresponding SAED pattern, (**d**) HAADF-STEM image of AB-type 2D BNSLs. (**c**) Structural model of AB-type 2D BNSLs. (**e**) GISAXS pattern for AB-type 2D BNSLs taken at an incidence angle of 0.2° with 10 keV X-rays. (**f**) Horizontal line profile of the GISAXS pattern along *q*_*z*_=0.047±0.003 Å^−1^. (**g**,**h**) HAADF-STEM tomographic reconstruction of AB-type 2D BNSLs; **g** is a perspective image of a 3D representation of a subset of the reconstruction and **h** is a cross-sectional slice showing that the NCs composing the 2D BNSL are coplanar. Scale bars, (**a**) 100 nm; (**d**) 20 nm; (**h**) 50 nm.

When the total NC concentration was reduced by 10–15 times in the spreading solution, 2D BNSLs were formed over extend areas (centimeter scale). Figure 5a shows an example of AB-type 2D BNSLs self-assembled from 3.8 nm (3.0 k) Au and 13.4 nm (5.3 k) Fe_3_O_4_ NCs. The SAED pattern (Fig. 5b), HAADF-STEM image (Fig. 5d) and SEM images (Supplementary Fig. 14) confirm that the superlattice is a monolayer in thickness. The large Fe_3_O_4_ NCs arranged to form a square lattice, with the smaller Au NCs occupying the centre of individual squares, which is essentially the 2D analogue of the CsCl-type lattice^67^. The difference in electron density between the two NC cores is clearly visible from the HAADF-STEM image, where the dark background is mostly filled with polystyrene brushes (Fig. 5d). Importantly, the same mixture of Au and Fe_3_O_4_ NCs would have become phase-separated or formed disordered films without polystyrene ligands. To complement electron microscopy studies and to probe quantitatively the BNSL structure and the degree of ordering over statistically relevant areas (mm^2^), we carried out grazing incidence small-angle X-ray scattering (GISAXS) measurements on the AB-type 2D BNSLs. As shown in Fig. 5e, the rod-like shape of the intense scattering features (Bragg rods) in the 2D GISAXS image suggests the presence of an ordered NC monolayer. The scattering profile along the *q*_*x*_ direction corresponding to in-plane ordering features three orders of diffraction peaks spaced as 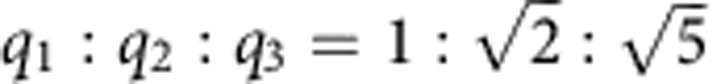 (Fig. 5f). These three peaks can be indexed as the (11), (20) and (31) reflections of a 2D face-centred square lattice. The absence of the (22) reflection may be due to its coincidence with a form factor minimum. The spacing calculated from the first-order diffraction peak by using the formula 
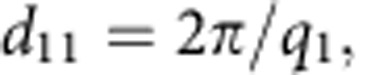
 is 17.8 nm, which is in good agreement with the average centre-to-centre distance between nearest-neighbour Fe_3_O_4_ NCs determined from TEM (18.3±1.0 nm). Therefore, the GISAXS results confirm that AB-type 2D BNSLs are phase-pure over macroscopic length scales as they appear under electron microscopy surveys. To reveal whether the Au and Fe_3_O_4_ NCs of AB-type BNSLs are coplanar, we further performed HAADF-STEM electron tomography (Fig. 5g and Supplementary Movie 1). The cross-sectional slice of the 3D tomographic reconstruction of the AB-type BNSLs clearly shows coplanarity of the two NC building blocks (Fig. 5h). When the concentration ratio between 3.8 nm (3.0 k) Au and 13.4 nm (5.3 k) Fe_3_O_4_ NCs was increased, A_2_B_3_-type and AB_8_-type 2D BNSLs were obtained (Supplementary Fig. 15). Notably, the AB_8_ structure has not been reported in previous works on 2D BNSLs^68^.

## Discussion

We have demonstrated the possibility of forming long-range-ordered binary superlattices using spherical polymer-grafted NCs. A rational choice of polymer-binding group and an optimized ligand-exchange protocol enable attachment of polymer brushes onto NCs of several different sizes and compositions with high grafting densities (>0.5 nm^−2^). We determined that the brush thickness *h* scales roughly as the square root of the molecular weight *M*_*n*_ of the polymer chains in the melt, and as 
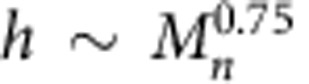
when dispersed in good solvents. The scaling behaviour of the brush thickness with molecular weight is in excellent agreement with the existing theoretical predictions and establishes a practical ‘calibration curve' for synthesizing modular polymer-NC building blocks with controlled effective size and interaction potential. A rich variety of 2D and 3D BNSL structures can be accessed from these NCs, most of which agree reasonably well with predictions based on maximized packing densities. The use of polymeric ligands provides quantitative and precise control over nearest-neighbour interparticle distances ranging from several to several tens of nanometres within complex 3D NC assemblies. This is difficult, if not impossible, with the short alkyl-chain-based ligands that have been used in previous studies. Our results open up new and exciting opportunities for the modular design of multicomponent blends of polymer-NC composites for the creation of mechanically robust inorganic–organic materials with controlled nanoscale interfaces and microstructures.

## Methods

### NC synthesis

All NC syntheses were carried out under argon atmosphere using standard Schlenk line techniques. Au NCs (3.8 and 4.2 nm)^40^, Au NCs (4.5 nm, 6.1 and 7.6 nm)^41^, and Fe_3_O_4_ NCs^42^ were synthesized according to literature methods.

### NC ligand exchange

Polystyrene-based ligands (Polymer Source, Inc.) were attached to the NC surface by a simple ligand-exchange reaction. Thiol-terminated and carboxyl-terminated polystyrenes were used as the polymeric ligand for Au and Fe_3_O_4_ NCs, respectively. In a typical process, as-synthesized Au or Fe_3_O_4_ NCs were dissolved into 5 ml of tetrahydrofuran (THF) at a NC concentration of 5 mg ml^−1^. Another 5 ml THF solution of end-functionalized polystyrene was prepared separately with a polymer concentration of 40 mg ml^−1^. The polystyrene solution was then added dropwise under sonication to the NC solution, and the resulting mixture was left to stir for 24 h at room temperature to promote surface saturation with polymeric ligands. The polymer-modified NCs were isolated by ethanol precipitation and centrifugation at 7,000 r.p.m. for 3 min. The supernatant was removed and the NCs were redispersed in THF. The above solvent–nonsolvent cleaning procedure was repeated 4–6 times to remove excess unbound polymers. The NCs were finally dispersed in toluene to a concentration of about 3 mg ml^−1^.

### Self-assembly of NC superlattices

BNSLs were formed by co-crystallization of a binary mixture of NCs on top of an immiscible liquid subphase at room temperature. In a typical process, a toluene solution (∼15 μl) containing two types of NCs was drop-cast onto the surface of DEG in a Teflon well (1.5 × 1.5 × 1.5 cm^3^). The well was then covered with a glass slide to slow down the evaporation rate of toluene. After 5 h, the BNSL film was transferred onto a carbon-coated Cu TEM grid (300-mesh) or a piece of Si wafer (1 × 1 cm^2^), which was further dried under vacuum for 30 min to remove residual DEG. Crystal structure models were built using the VESTA software package (version 3.1.8)^69^.

### Statistical analysis of interparticle distances from TEM images

Using the ImageJ software, experimental TEM images of 2D single-component hexagonal superlattices were first converted to binary images and the centre of mass of individual particles were determined. Delaunay triangulation then generates a set of bonds connecting nearest-neighbour particle centres. A large number of bond counts (usually between 1,000 and 5,000) were obtained by analysing many TEM images acquired from different areas of the same sample (Supplementary Fig. 6). The distribution of bond length (that is, interparticle distance) was plotted using OriginPro 8.0.

### Electron microscopies and GISAXS

Bright-field TEM images and SAED patterns were acquired on a 200-kV Tecnai G2 20 S-TWIN TEM equipped with a Gatan Orius SC200 CCD camera. SAED patterns were collected from areas of about 0.5 μm^2^. HAADF-STEM images were taken on a 200-kV FEI monochromated F20 UT Tecnai TEM. SEM imaging was carried out on a Zeiss Gemini Ultra-55 microscope operating at 5 kV. GISAXS experiments were conducted at beamline 7.3.3 at the Advanced Light Source at Lawrence Berkeley National Laboratory. X-rays (10 keV) with the energy bandwidth 
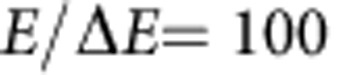
 were used. The beam size was about 300 μm vertically and 700 μm horizontally. A Pilatus 2M detector (pixel size: ∼172 μm) from Dectris was positioned 3.8 m away from the sample. The reciprocal space calibration was performed by using the silver behenate standard. Samples for GISAXS measurements were deposited onto Si substrates. A series of incident angles ranging from 0.05° to 0.25° was used for each sample. GISAXS results presented here were obtained with an incidence angle of 0.20°, which is greater than the critical angle of Si (0.18°) and thus ensures full penetration of X-rays into the film. Image processing was done using the Igor NIKA software package. The scattering intensities (*I*) are plotted with respect to wavevector *q*, where *q*=(4*π/λ*)* *sin* θ*, *λ* is the wavelength of the incident X-ray beam, and 2*θ* is the scattering angle.

### Electron tomography

The projection images for 3D electron tomography were acquired using an FEI Titan 80/300 operated in HAADF-STEM mode at 200 kV with a 10 mrad probe semi-convergence angle. The pixel size was 2.27 nm. HAADF-STEM images were taken from −65° to 72° to create a tilt series of 78 images. The tilt series was aligned and reconstructed using the eTomo software of the IMOD tomography package. Post-processing was performed in Matlab, and 3D visualization was performed using Tomviz 0.6.1.

### TGA, DLS and FTIR

TGA was performed by using a Q5000 system (TA Instruments). The sample (5–10 mg) was loaded into an aluminium pan and was heated to 600 °C at a constant heating rate of 20 °C min^−1^ under either argon (for polystyrene alone or polystyrene-grafted NCs) or air (for as-synthesized NCs or oleylamine alone). Analysis was carried out using the TA Universal Analysis software package. The ligand grafting densities (*σ*) were estimated by using the formula: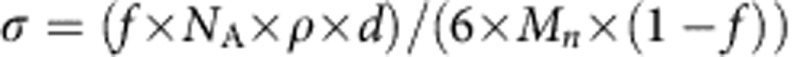, under the assumptions that the density of the spherical NCs is identical to the density of the bulk material and that no free polymer is present. Here, *f* is the weight fraction of the organic ligands determined by TGA, *N*_A_ is the Avogadro constant, *ρ* is the bulk density of NC's core material (19.32 g cm^−3^ for Au and 5.15 g cm^−3^ for Fe_3_O_4_), *d* is the diameter of NC's core and *M*_*n*_ is the number-average molecular weight of the polymeric ligands. FTIR spectra were taken in transmission mode on a Thermal Scientific Nicolet 6700 spectrometer equipped with a deuterated triglycine sulfate detector. Samples for FTIR measurements were prepared by drop-casting concentrated NC solutions onto a KBr substrate (International Crystal Laboratories) followed by drying under vacuum. DLS measurements were carried out on a Zetasizer Nano-ZS system (Malvern), equipped with a He–Ne 633 nm laser as the light source. The as-synthesized NCs were dispersed in hexane and polystyrene-capped NCs were dispersed in toluene. Samples were filtered through a 0.2-μm polytetrafluoroethylene syringe filter and were filled in a quartz cuvette for DLS measurements. The hydrodynamic diameter *D*_h_ was determined from the Stokes–Einstein equation, *D*_h_*=k*_B_*T/3πηD*_dif_, where *D*_dif_ is the diffusion coefficient of NCs, *T* is temperature, *k*_B_ is the Boltzmann constant and *η* is solvent viscosity. *D*_dif_ can be obtained from the intensity correlation function. Hydrodynamic diameters, reported as number averaged values, are averages of five measurements each composed of 15 scans.

## Additional information

**How to cite this article:** Ye, X. *et al.* Structural diversity in binary superlattices self-assembled from polymer-grafted nanocrystals. *Nat. Commun.* 6:10052 doi: 10.1038/ncomms10052 (2015).

## Supplementary Material

Supplementary Information Supplementary Figures 1-15, Supplementary Tables 1-6 and Supplementary References

Supplementary Movie 1 Tomography reconstruction of AB-type two-dimensional binary nanocrystal superlattices

## Figures and Tables

**Table 1:** Grafting densities (chains nm^−2^) of polystyrene ligands on Au NCs estimated from TGA.

**Core diameter**	**OLAM**	**PS-SH (1.1 k)**	**PS-SH (1.5 k)**	**PS-SH** **(3.0 k)**	**PS-SH (5.0 k)**	**PS-SH (11.5 k)**	**PS-SH (20.0 k)**
3.8 nm Au	5.60	2.62	—	2.25	1.71	1.08	—
4.5 nm Au	5.75	2.16	—	2.28	1.89	—	—
7.6 nm Au	4.92	—	2.39	1.79	1.27	0.95	0.83

OLAM, oleylamine; PS-SH, thiol-terminated polystyrene; TGA, thermal gravimetric analysis.

The estimated error is less than 10% based on multiple runs with the same sample.
