# Supplementary material for: Structural diversity in binary superlattices self-assembled from polymer-grafted nanocrystals
Source: Nat Commun. 2015 Dec 2;6:10052. doi: 10.1038/ncomms10052 (PMC4686769; doi:10.1038/ncomms10052)
Supplement: Supplementary Information — Supplementary Figures 1-15, Supplementary Tables 1-6 and Supplementary References [file ncomms10052-s1.pdf]

Supplementary Figures

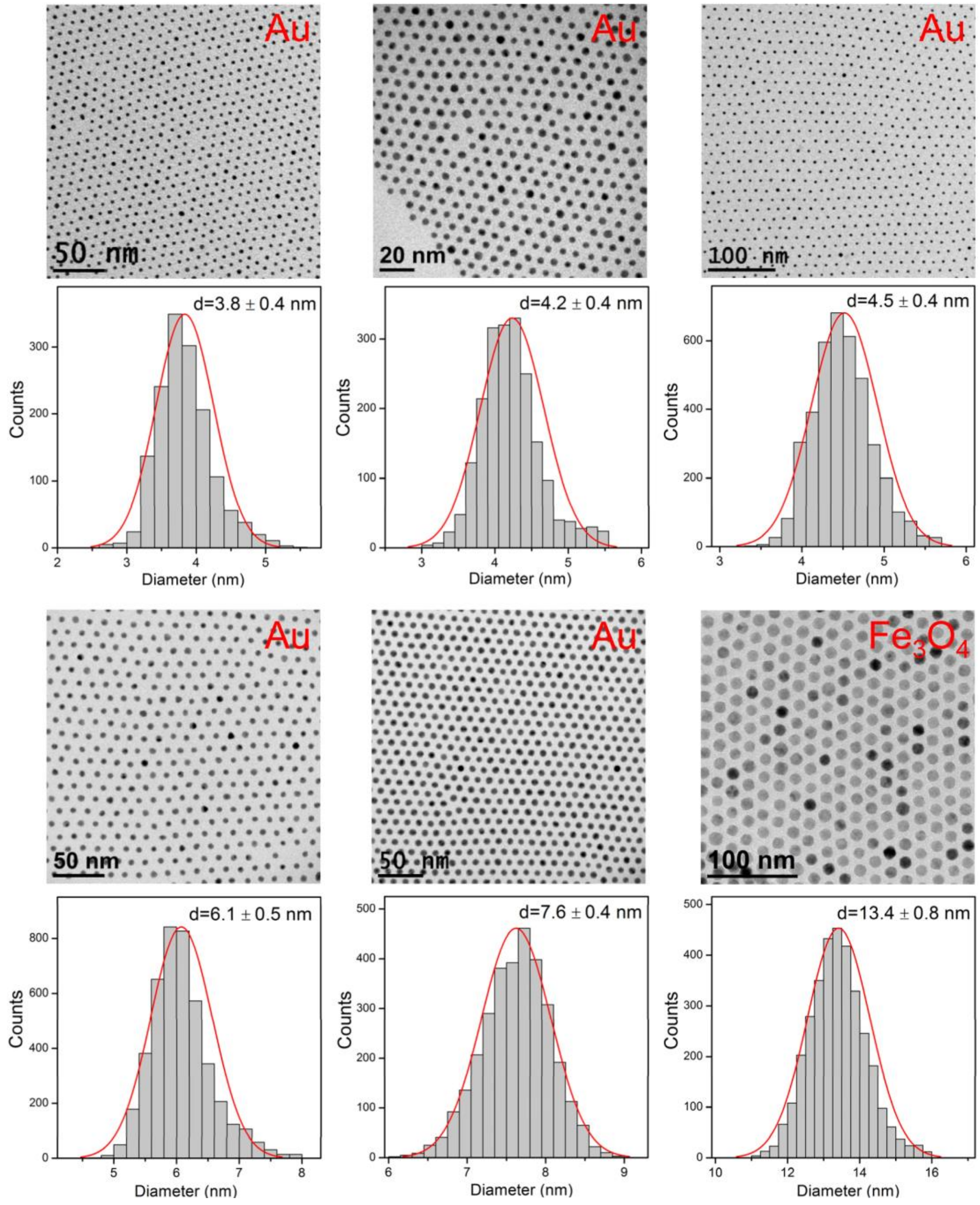

Supplementary Figure 1 | Determination of NC core size. Collection of representative TEM images of differently sized Au and Fe_3O_4 NCs and corresponding NC size distribution histogram obtained from TEM image analysis. The red lines represent the Gaussian fit to the size distribution.

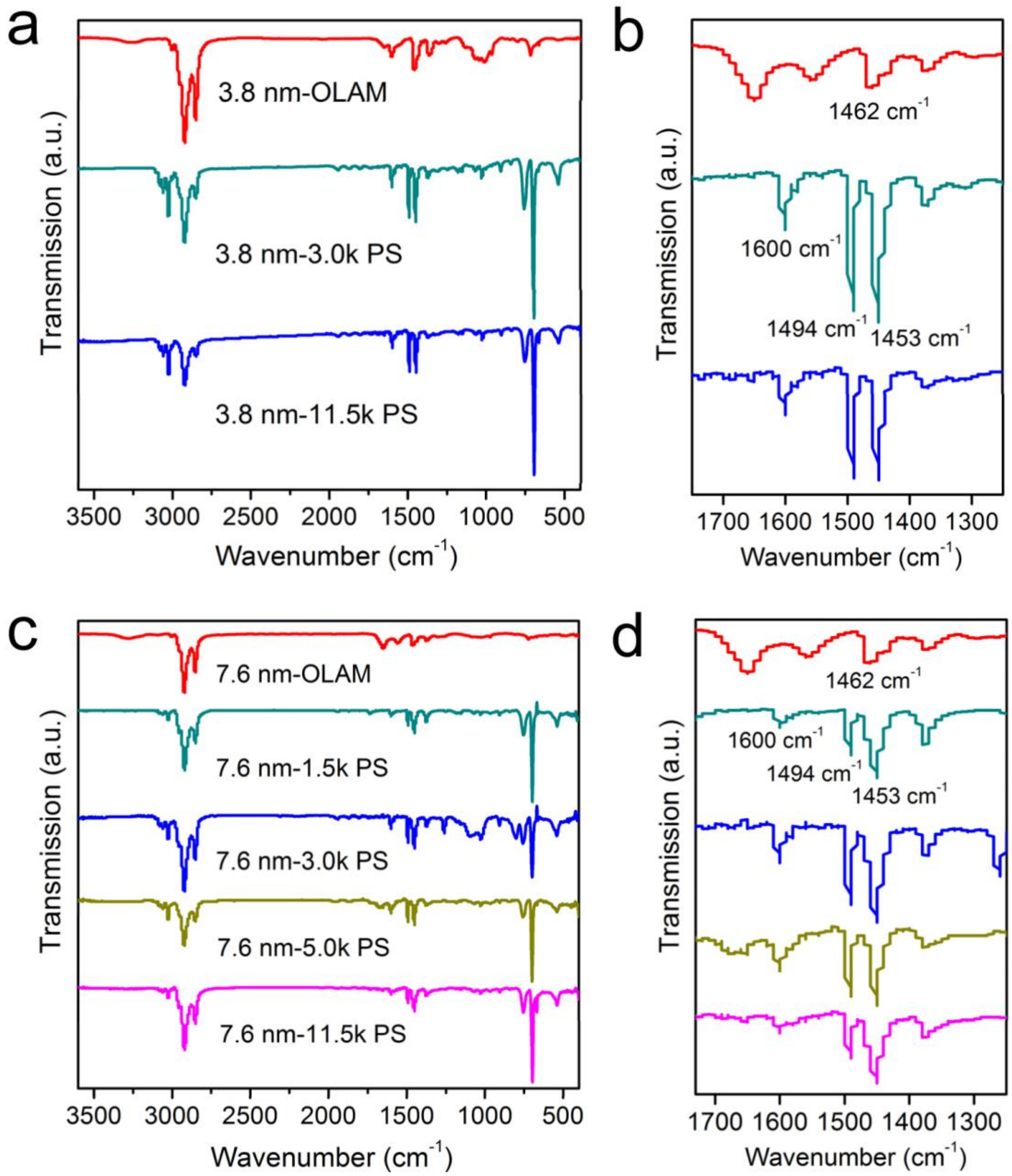

Supplementary Figure 2 | FTIR characterization. FTIR spectra of as-synthesized oleylamine-capped (**a**) 3.8 nm and (**c**) 7.6 nm Au NCs. (**b**) and (**d**) are expanded views in the spectra regions of 1730-1150 cm^{-1} for the FTIR spectra shown in (**a**) and (**c**), respectively. The spectra have been vertically offset for clarity purposes. The absorption peak at 1600 cm^{-1} arises from the C=C stretching of the aromatic ring of polystyrene, and the absorption peaks at 1494 and 1453 cm^{-1} are C-H bending modes of the aromatic ring of polystyrene.[1] These distinct spectroscopic features were not observed for oleylamine-capped NCs (red curves).

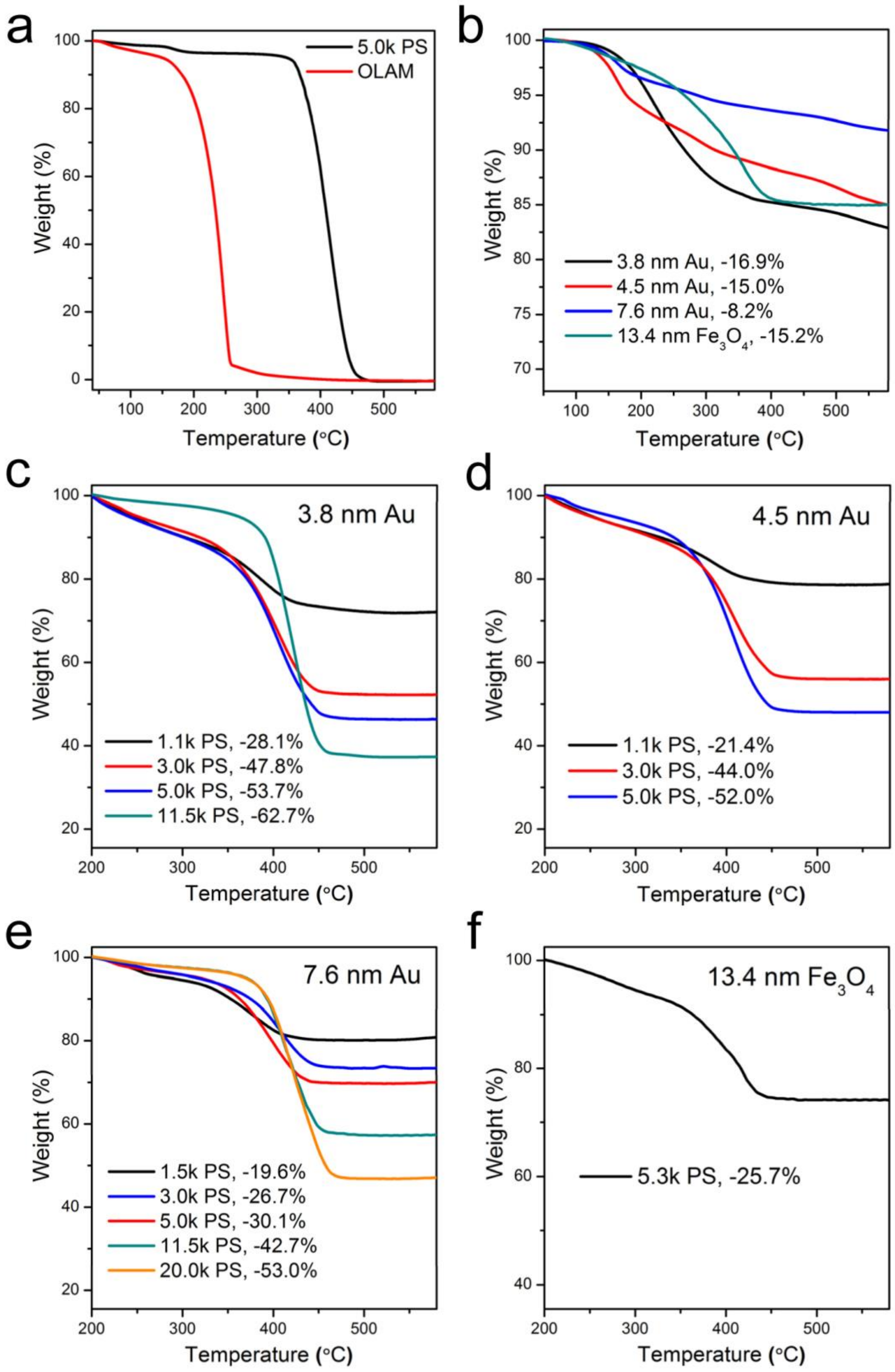

Supplementary Figure 3 | TGA characterization. (**a**) TGA results of oleylamine and thiol-terminated polystyrene with *Mn*=5 x 10^3 g mol^{-1}. (**b**) TGA results of as-synthesized Au and Fe_3O_4 NCs. (**c-f**) TGA results of polystyrene functionalized (**c-e**) Au and (**f**) Fe_3O_4 NCs. The mass losses are indicated for individual traces on the graph. The grafting density for the 13.4 nm Fe_3O_4 NCs is estimated to be 0.45 chains nm^{-2}, while those for various Au NCs are presented in Table 1 in the maintext.

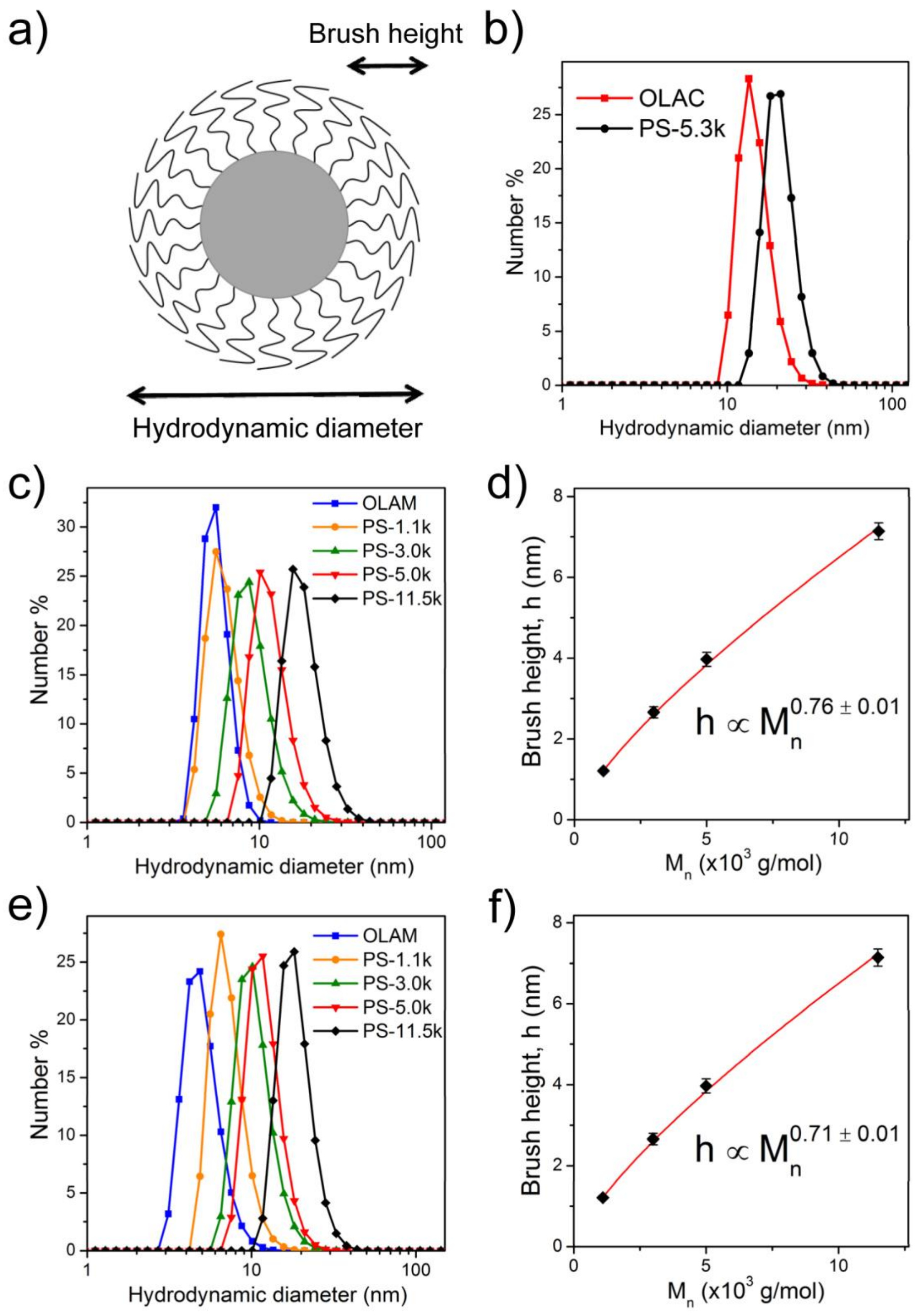

Supplementary Figure 4 | DLS results and scaling relations between polymer brush height and molecular weight. (**a**) Schematic illustration of NCs end-grafted with polymer brushes in good solvents. (**b, c, e**) Number averaged NC size distribution from DLS measurements for (**b**) 13.4 nm Fe_3O_4, (**c**) 3.8 nm Au and (**e**) 4.5 nm Au NCs. (**d, f**) Scaling relations of DLS-derived brush height, h, as a function of the molecular weight of thiol-terminated polystyrene ligands for (**d**) 3.8 nm and (**f**) 4.5 nm Au NCs dispersed in toluene. The red curves represent the best fit to the data using power-law functions.

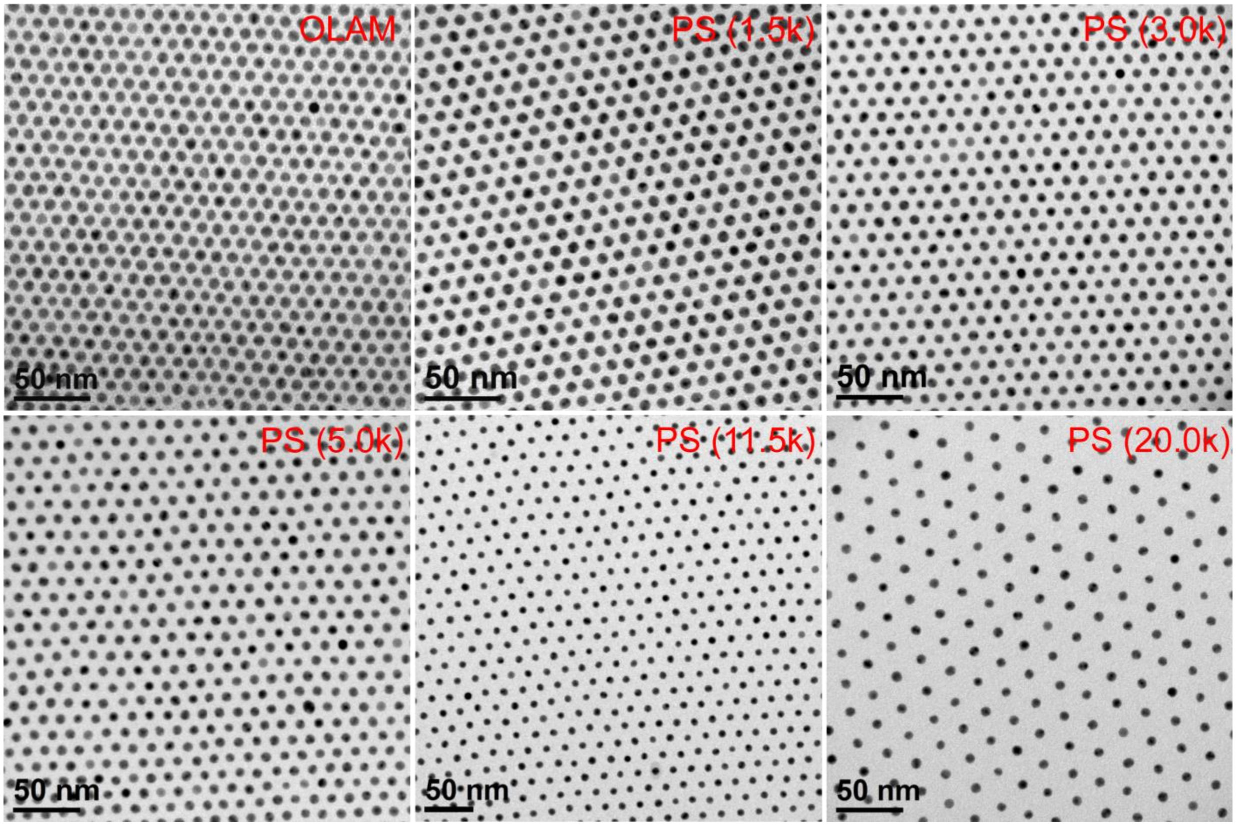

Supplementary Figure 5 | Representative TEM images of OLAM-capped and polystyrene-grafted 7.6 nm Au NCs. The molecular weights of the polystyrene ligands are indicated on the images.

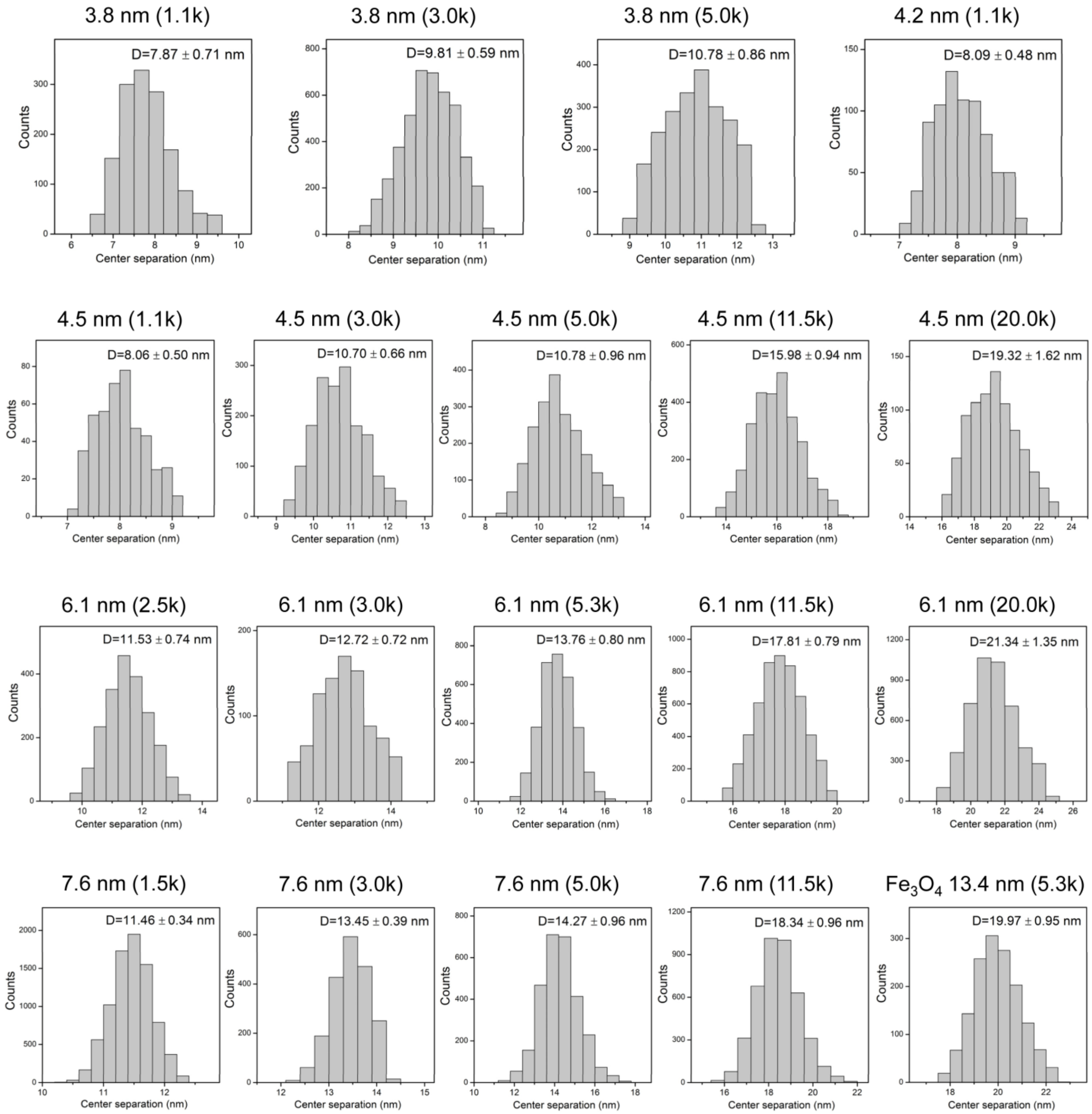

Supplementary Figure 6 | Histogram of nearest-neighbor center-to-center interparticle distance *D* obtained from particle tracking analysis of TEM images of two-dimensional NC superlattices. The inorganic core size and the molecular weight of the polystyrene ligands are indicated on top of each graph.

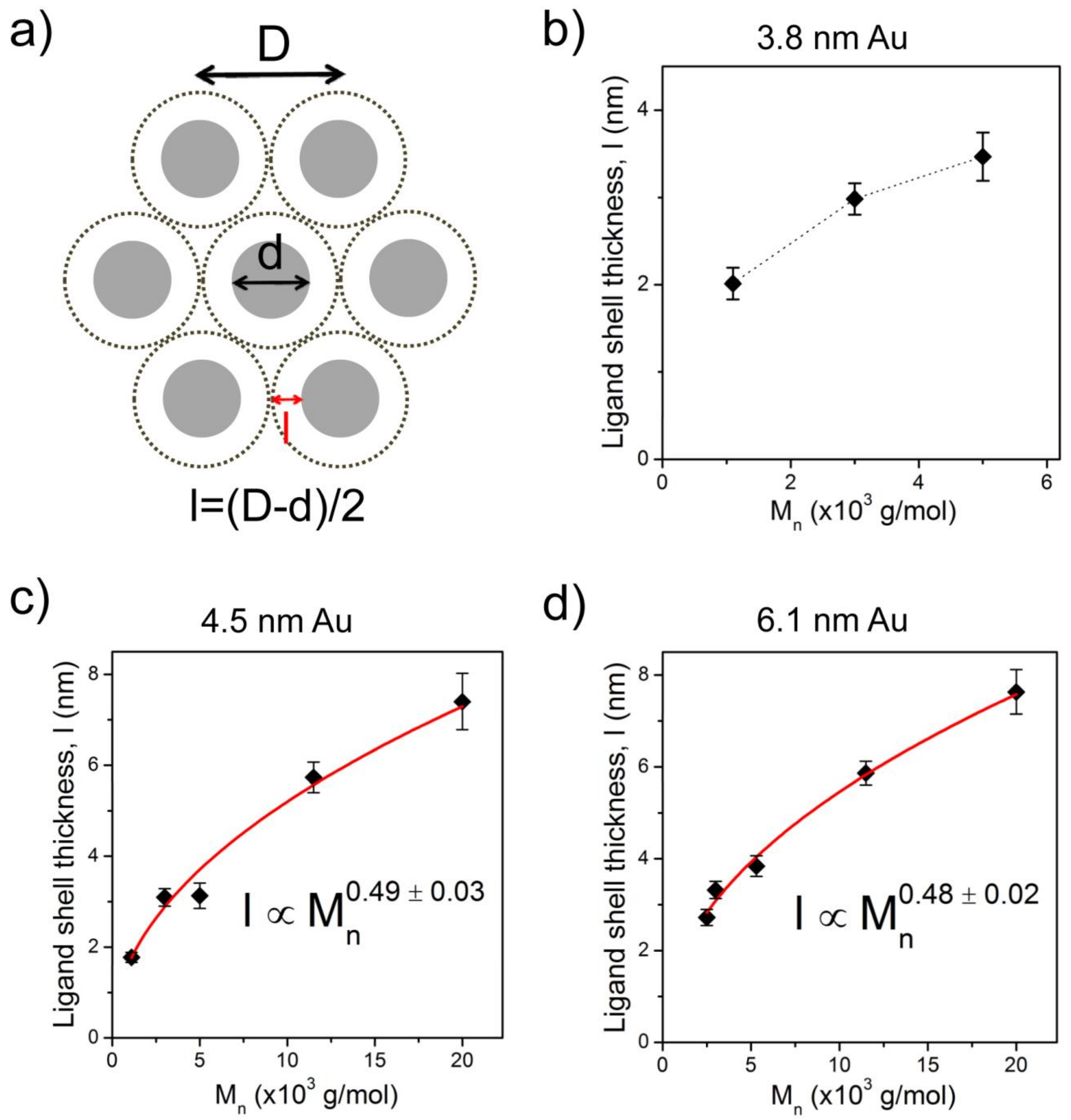

Supplementary Figure 7 | Scaling relations between ligand shell thickness and molecular weights of polymer brushes. (**a**) Schematics showing the relation between *D*, the center-to-center distance between nearest-neighbor NCs, *d*, the diameter of the inorganic core of NCs, and the ligand shell thickness, *l=(D-d)/2*, for two-dimensional hexagonal NC superlattices. (**b-d**) Scaling relations of ligand shell thickness *l* as a function of molecular weight for (**b**) 3.8 nm Au, (**c**) 4.5 nm Au and (**d**) 6.1 nm Au NCs. The red curves in (**c**) and (**d**) represent the best fit to the data using power-law functions.

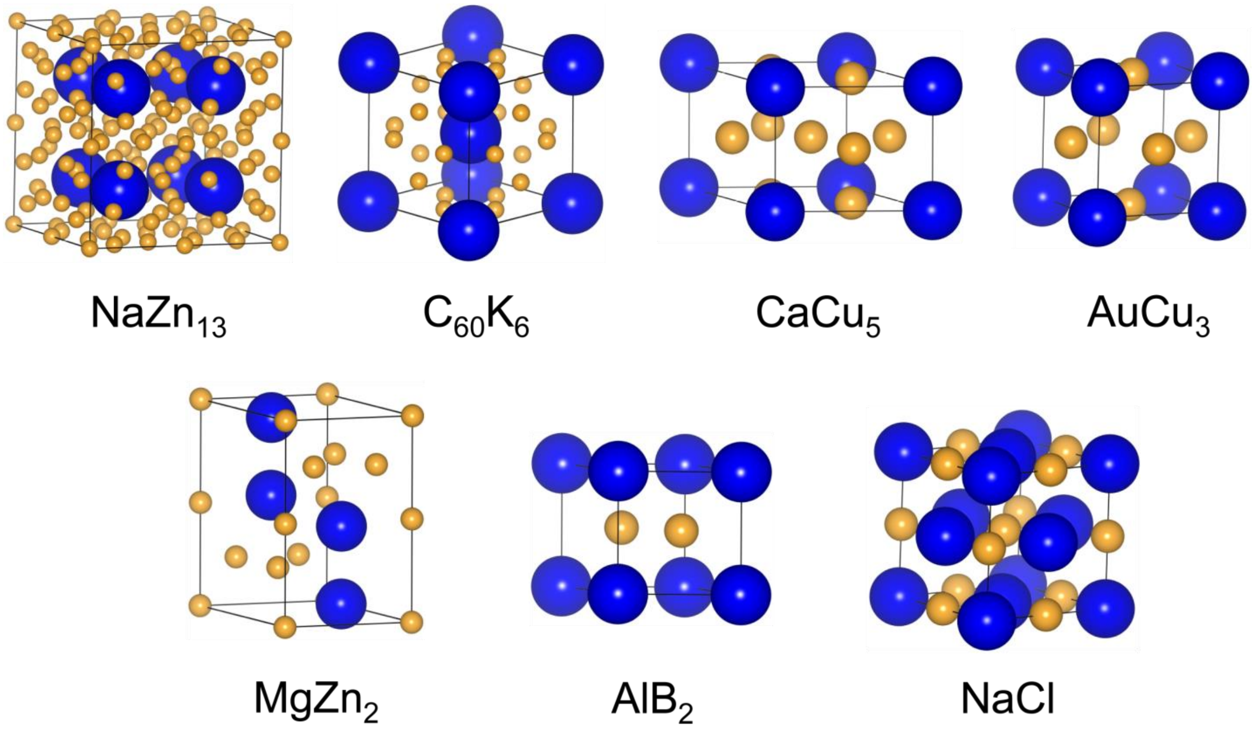

Supplementary Figure 8 | Structural models of the unit cell of different BNSL phases reported in this work.

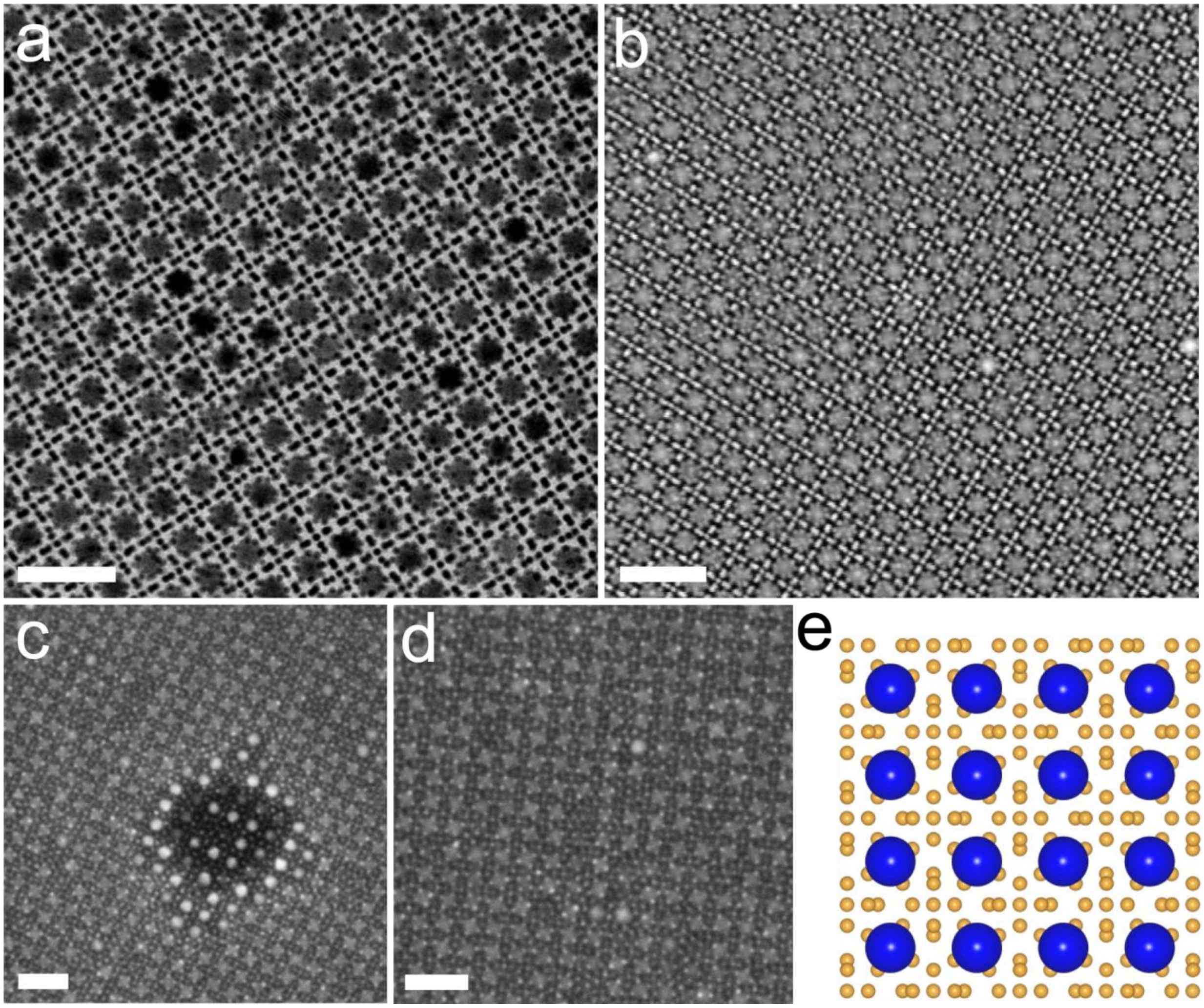

Supplementary Figure 9 | Structural characterization of $NaZn_{13}$-type BNSLs. (**a**) TEM, (**b**) STEM-HAADF and **(c,d**) SEM images of $NaZn_{13}$-type BNSLs self-assembled from 3.8 nm (3.0 k) Au and 13.4 nm (5.3 k) Fe_3O_4 NCs. (**e**) Structural model of the [001] projection of $NaZn_{13}$-type BNSLs. All scale bars represent 50 nm.

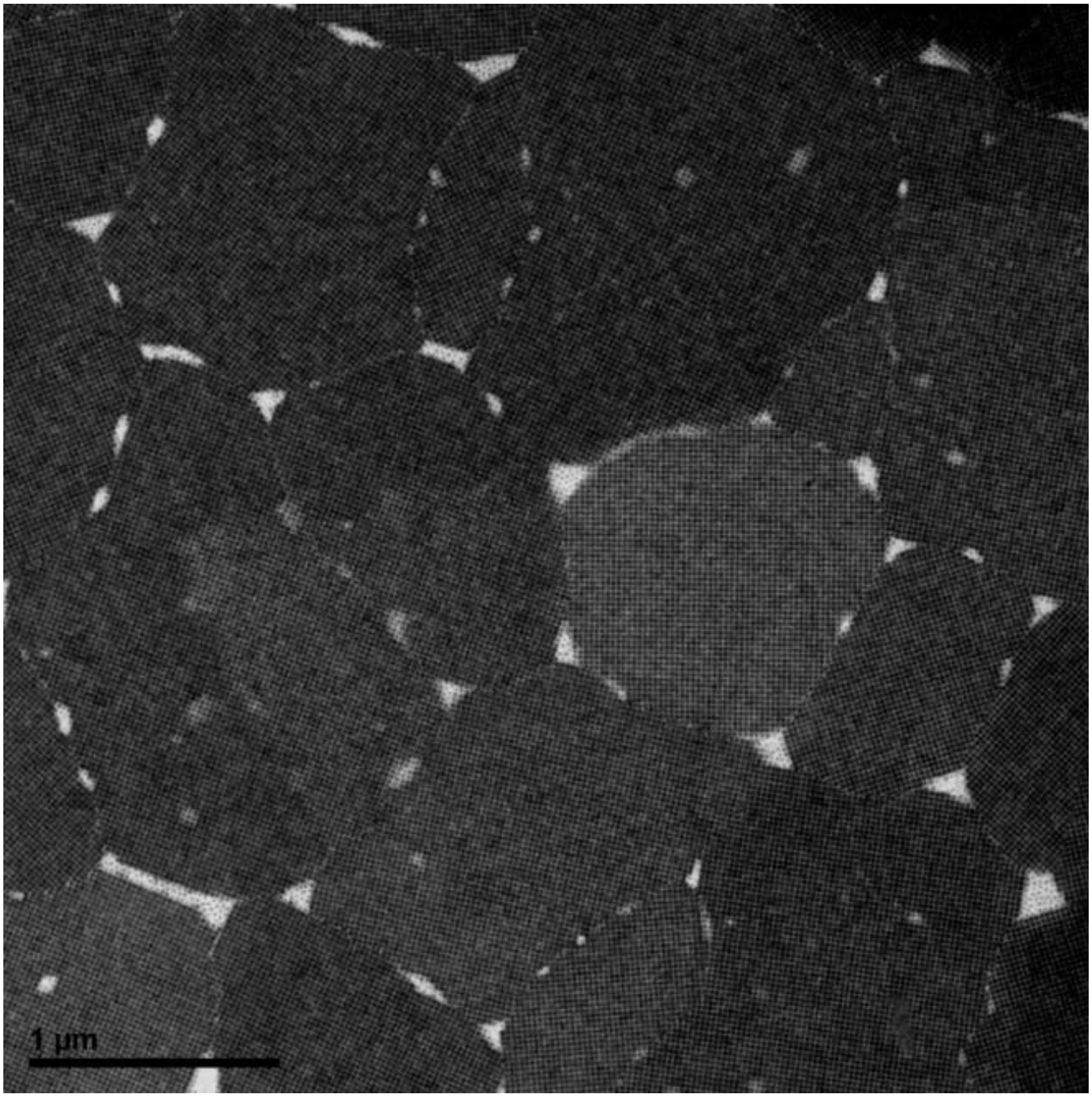

Supplementary Figure 10 | Structural characterization of $NaZn_{13}$-type BNSLs. Representative low-magnification TEM image of $NaZn_{13}$-type BNSLs self-assembled from 3.8 nm (3.0 k) Au and 13.4 nm (5.3 k) Fe_3O_4 NCs.

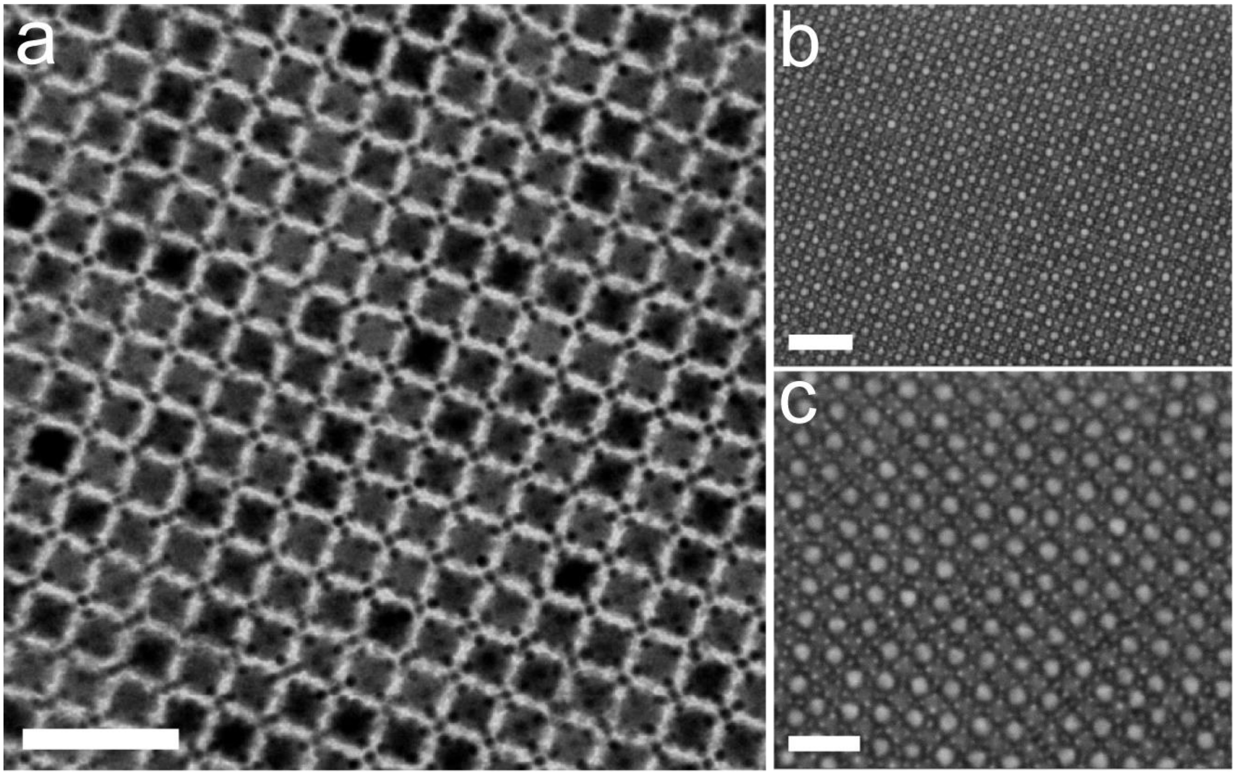

Supplementary Figure 11 | Structural characterization of bcc-AB_6-type BNSLs. Additional (**a**) TEM and (**b,c**) SEM images of bcc-AB_6-type (isostructural with the K_6C_{60} phase) BNSLs self-assembled from 3.8 nm (3.0 k) Au and 13.4 nm (5.3 k) Fe_3O_4 NCs. Scale bars: (**a**) 50 nm, (**b**) 100 nm, (**c**) 50 nm.

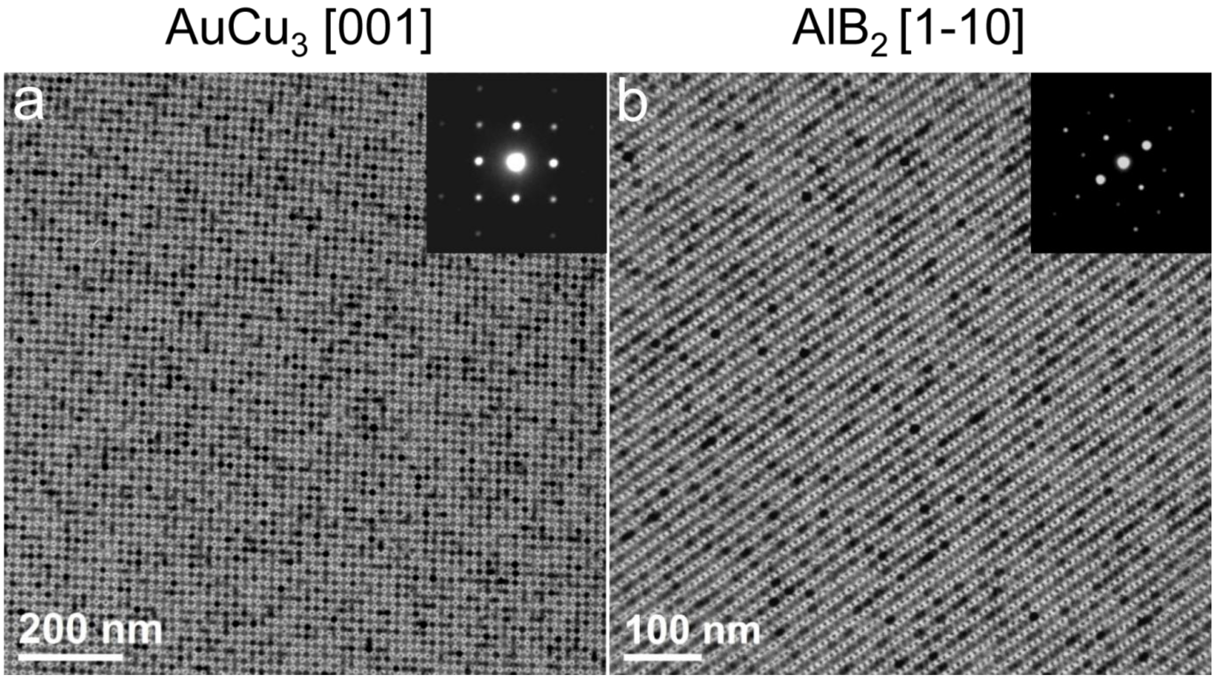

Supplementary Figure 12 | Structural characterization of $AuCu_3$- and AlB_2-type BNSLs. Low-magnification TEM image and corresponding small-angle electron diffraction (SAED) pattern of (**a**) $AuCu_3$-type and (**b**) AlB_2-type BNSLs self-assembled from 3.8 nm (3.0 k) Au and 13.4 nm (5.3 k) Fe_3O_4 NCs.

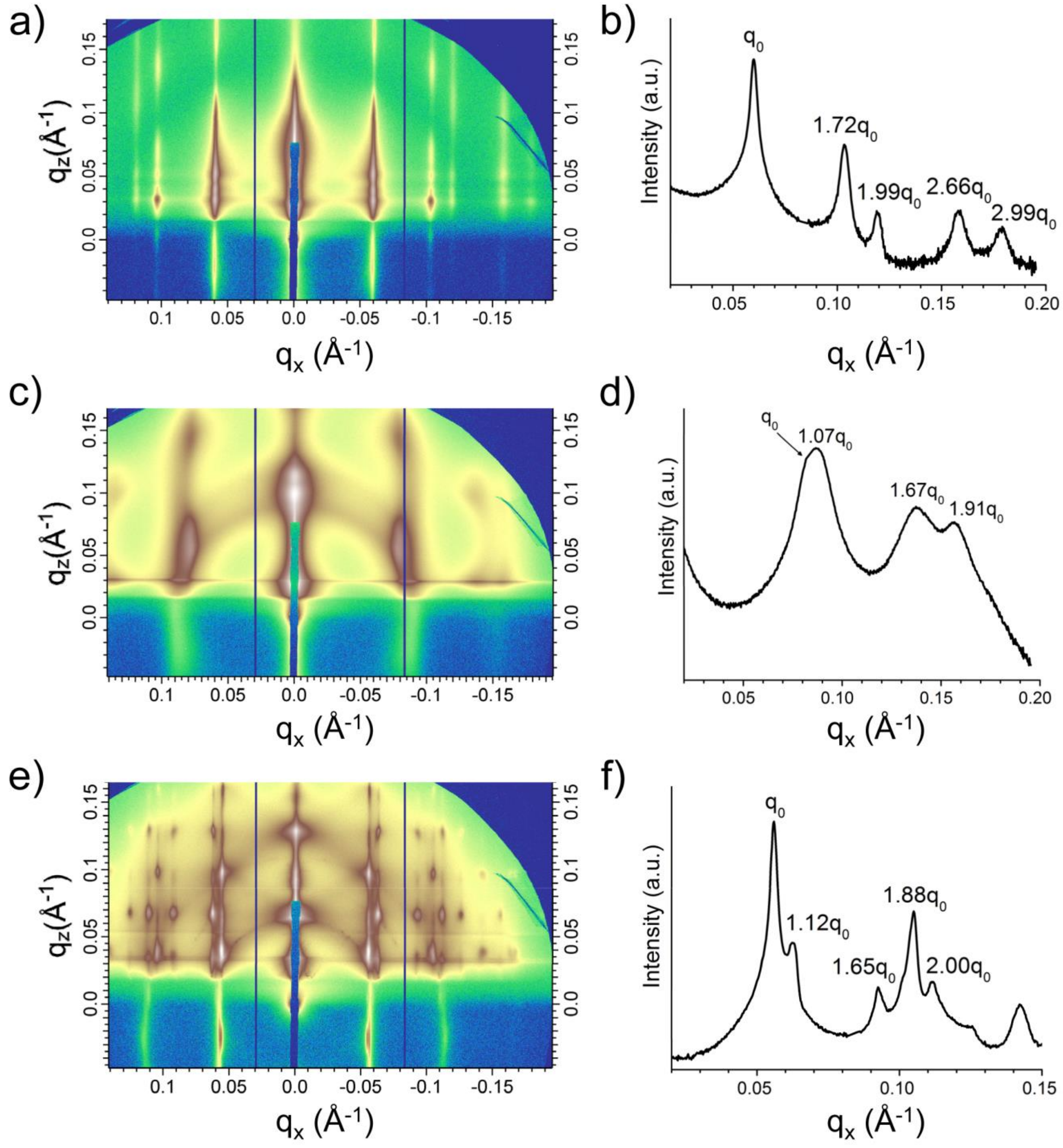

Supplementary Figure 13 | GISAXS characterization of nanocrystal superlattices. (**a, c, e**) GISAXS patterns and (**b,d,f**) corresponding horizontal line profiles for NC superlattices self-assembled on the DEG surface using (**a,b**) 7.6 nm (3.0 k) Au, (**c,d**) 3.8 nm (3.0 k) Au and (**e,f**) 4.5 nm (11.5 k) Au. All GISAXS patterns were taken at an incidence angle of 0.2° with 10 keV X-rays. The horizontal line profiles are taken along $q_z = 0.032 \pm 0.002$ Å^{-1} (**b**), $q_z = 0.029 \pm 0.002$ Å^{-1} (**d**) and $q_z = 0.037 \pm 0.002$ Å^{-1} (**f**), respectively.

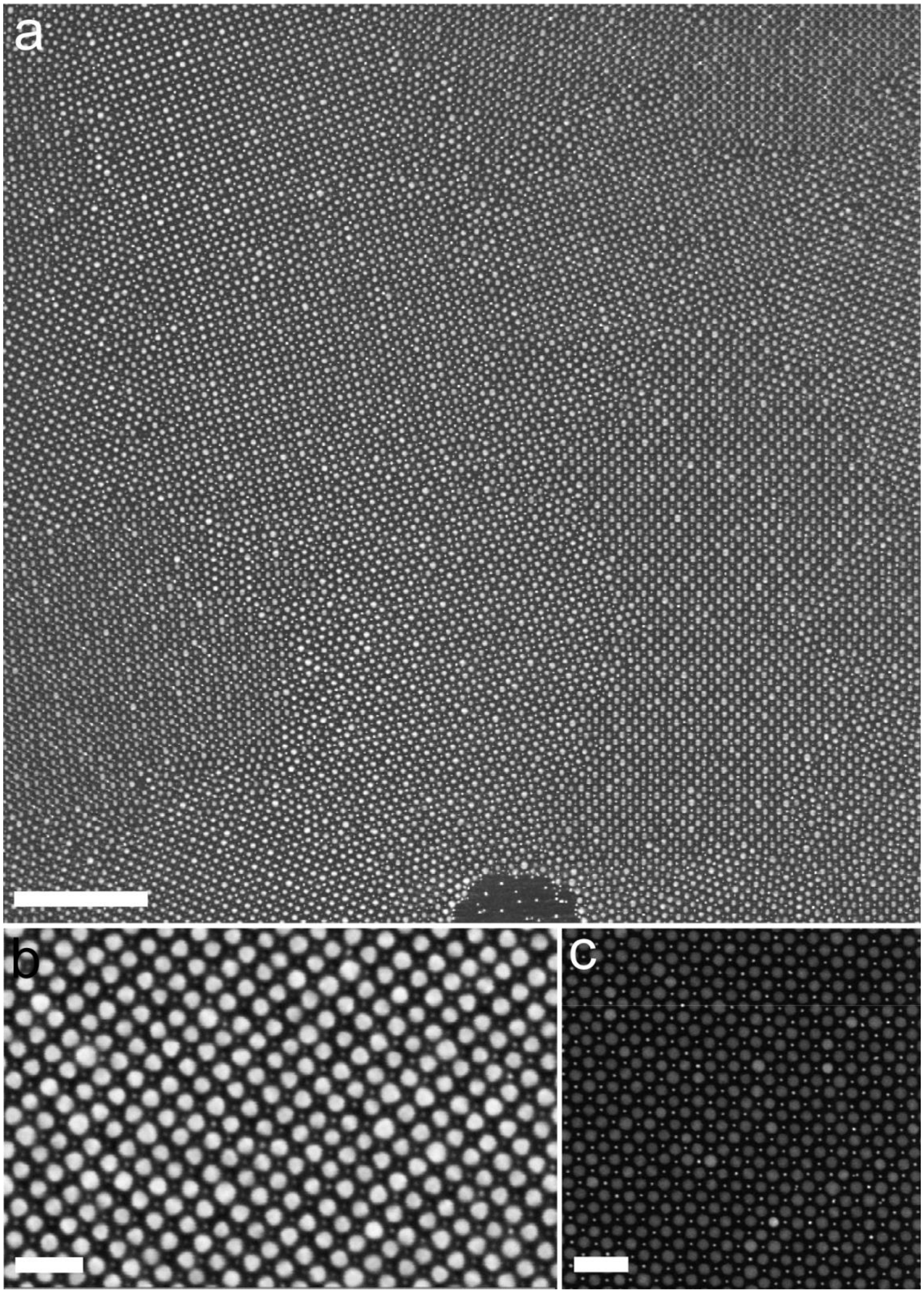

Supplementary Figure 14 | HAADF-STEM tomography and electron microscopy characterization of AB-type 2D BNSLs. (**a**) A single slice of a 3D tomographic reconstruction of AB-type 2D BNSLs. (**b**) High-magnification SEM images and (**c**) STEM-HAADF image of AB-type two-dimensional (2D) BNSLs self-assembled from 3.8 nm (3.0 k) Au and 13.4 nm (5.3 k) Fe_3O_4 NCs. Scale bars: (**a**) 250 nm, (**b**) 50 nm, (**c**) 50 nm.

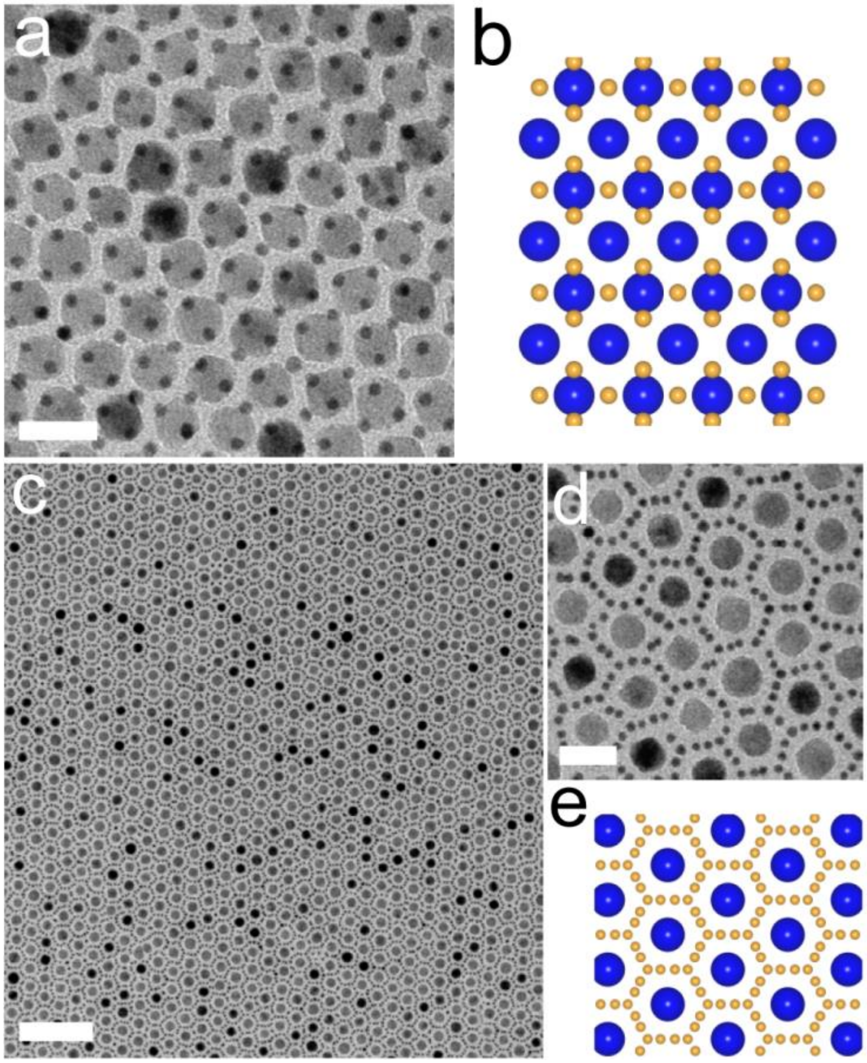

Supplementary Figure 15 | Structural diversity in 2D BNSLs. (**a**) TEM image of A_2B_3-type 2D BNSLs self-assembled from 3.8 nm (3.0k) Au and 13.4 nm (5.3k) Fe_3O_4 NCs. (**b**) Structural model of A_2B_3-type 2D BNSLs. (**c**) Low-magnification and (**d**) high-magnification TEM images of AB_8-type 2D BNSLs self-assembled from 3.8 nm (3.0k) Au and 13.4 nm (5.3k) Fe_3O_4 NCs. (**e**) Structural model of AB_8-type 2D BNSLs. Scale bars: (**a**) 20 nm, (**c**) 100 nm, (**d**) 20 nm.

Supplementary Tables

Supplementary Table 1. Number-average molecular weight M_n and polydispersity index (PDI) of polystyrene-based end-functionalized polymers, as provided by the manufacturer, Polymer Source, Inc.

Catalog number	M_n (x10^3 g/mol)	PDI
P10827-SSH	1.1	1.25
P2996-SSH	1.5	1.10
P18812-SSH	2.5	1.05
P8665-SSH	3.0	1.07
P4429-SSH	5.0	1.40
P4430-SSH	5.3	1.10
P8724-SSH	11.5	1.08
P8659-SSH	20.0	1.07
P18049-SCOOH	5.3	1.04

Supplementary Table 2. Average distance between adjacent polystyrene tethers on the surface of Au NCs and the Flory radius of polystyrene chains with the same molecular weights.

Core diameter	OLAM	PS-SH (1.1k)	PS-SH (1.5k)	PS-SH (3.0k)	PS-SH (5.0k)	PS-SH (11.5k)	PS-SH (20.0k)
3.8 nm Au	0.46	0.68	--	0.73	0.84	1.06	--
4.5 nm Au	0.46	0.75	--	0.73	0.80	--	--
7.6 nm Au	0.50	--	0.71	0.82	0.98	1.13	1.21
R_F (nm)	--	2.32	2.80	4.24	5.76	9.49	13.23

The average distance (in nm) between adjacent polystyrene ligand grafting points on the surface of various Au NCs is estimated as $t = \frac{2}{\sqrt{\pi\sigma}} \cong \frac{1.1}{\sqrt{\sigma}}$, where σ is the ligand grafting density (chains nm^{-2}) estimated from TGA. The footprint of a single polystyrene molecule on the NC surface is modeled as a circular area with a diameter equal to the distance between adjacent chains. R_F: The Flory radius, defined as the root-mean-squared end-to-end distance for a free polymer chain. R_F values are calculated for polystyrene chains in a good solvent using the formula $R_F=b*N^{3/5}=b*(M_n/M_0)^{3/5}$, where b is the length of the Kuhn monomer (1.8 nm for polystyrene), N is the number of Kuhn segments, M_n is the number-average molecular weight, and M_0 is the molar mass of the Kuhn monomer (720 g mol^{-1} for polystyrene).[2]

Supplementary Table 3. Average hydrodynamic diameters D_h of polystyrene functionalized Au NCs determined from DLS measurements.

	PS-SH (1.1k)	PS-SH (1.5k)	PS-SH (2.5k)	PS-SH (3.0k)	PS-SH (5.0k)	PS-SH (5.3k)	PS-SH (11.5k)
3.8 nm Au	6.27	--	--	9.16	11.78	--	18.13
4.5 nm Au	7.19	--	--	10.44	12.33	--	18.65
6.1 nm Au	--	--	10.82	--	--	14.78	--
7.6 nm Au	--	9.86	--	14.47	15.65	--	22.83

Supplementary Table 4. Average DLS-derived brush height *h* for different polystyrene functionalized Au NCs.

	PS-SH (1.1k)	PS-SH (1.5k)	PS-SH (2.5k)	PS-SH (3.0k)	PS-SH (5.0k)	PS-SH (5.3k)	PS-SH (11.5k)
3.8 nm Au	1.21	--	--	2.66	3.97	--	7.14
4.5 nm Au	1.34	--	--	2.96	3.91	--	7.07
6.1 nm Au	--	--	2.37	--	--	4.35	--
7.6 nm Au	--	1.13	--	3.43	4.02	--	7.61

Supplementary Table 5. Average nearest-neighbor center-to-center interparticle distance *D* determined from particle tracking analysis of TEM images of two-dimensional NC superlattices.

	PS-SH (1.1k)	PS-SH (1.5k)	PS-SH (2.5k)	PS-SH (3.0k)	PS-SH (5.0k)	PS-SH (5.3k)	PS-SH (11.5k)	PS-SH (20.0k)
3.8 nm Au	7.87	--	--	9.81	10.78	--	--	--
4.2 nm Au	8.09	--	--	--	--	--	--	--
4.5 nm Au	8.06	--	--	10.7	10.78	--	15.98	19.32
6.1 nm Au	--	--	11.53	12.72	--	13.76	17.81	21.34
7.6 nm Au	--	11.46	--	13.45	14.27	--	18.34	--

Supplementary Table 6. Average ligand shell thickness *l= (D-d)/2*, determined from particle tracking analysis of TEM images of two-dimensional NC superlattices.

	PS-SH (1.1k)	PS-SH (1.5k)	PS-SH (2.5k)	PS-SH (3.0k)	PS-SH (5.0k)	PS-SH (5.3k)	PS-SH (11.5k)	PS-SH (20.0k)
3.8 nm Au	2.01	--	--	2.98	3.47	--	--	--
4.2 nm Au	1.93	--	--	--	--	--	--	--
4.5 nm Au	1.78	--	--	3.10	3.13	--	5.73	7.40
6.1 nm Au	--	--	2.72	3.32	--	3.84	5.86	7.63
7.6 nm Au	--	1.93	--	2.92	3.33	--	5.37	--
R_g (nm)	0.91	1.06	1.37	1.5	1.93	1.99	2.94	3.87

The radius of gyration R_g, of polystyrene, are calculated using the formula: $R_g = \frac{1}{\sqrt{6}} b * N = \frac{1}{\sqrt{6}} b * (\frac{M_n}{M_0})^{0.5}$, where b is the length of the Kuhn monomer (1.8 nm for polystyrene), N is the number of Kuhn segments, M_n is the number-average molecular weight, and M_0 is the molar mass of the Kuhn monomer (720 g mol^{-1} for polystyrene).[5]

Supplementary References

1. Liang, C. Y. & Krimm, S. Infrared spectra of high polymers. VI. Polystyrene. *J. Polym. Sci.* **27,** 241-254 (1958).

2. Rubinstein, M.; Colby, R. *Polymer physics*; Oxford University Press: New York. (2003).
